# Determination of the latent geometry of atorvastatin pharmacokinetics by transfer entropy to identify bottlenecks

**DOI:** 10.1186/s40360-025-00948-6

**Published:** 2025-06-25

**Authors:** Paola Lecca, Angela Re

**Affiliations:** 1 Faculty of Engineering, Free University of Bozen-Bolzano, NOI Techpark - via A. Volta 13/A, 39100 Bolzano-Bozen, Italy; 2 Member of the National Group for Mathematical Analysis, Probability and their Applications, Francesco Severi National Institute of High Mathematics, Città Universitaria - P.le Aldo Moro 5, 00185 Rome, Italy; 3 Department of Applied Science and Technology, Politecnico di Torino, Corso Duca degli Abruzzi 24, 10129 Turin, Italy

**Keywords:** Latent geometry, Transfer entropy, Graph embedding, Bottleneck analysis, Biochemical networks

## Abstract

**Background:**

In mathematics, a physical network (e.g. biological network, social network, IT network, communication network) is usually represented by a graph. The determination of the metric space (also referred to as latent geometry) of the graph and the disposition of its nodes on it provide important information on the reaction propensity and consequently on the possible presence of bottlenecks in a system of interacting molecules, such as it happens in pharmacokinetics. To determine the latent geometry and the coordinates of nodes, it is necessary to have the dissimilarity or distance matrix of the network, an input that is not always easy to measure in experiments.

**Results:**

The main result of this study is the mathematical and computational procedure for determining the distance/dissimilarity matrix between nodes and for identifying the latent network geometry from experimental time series of node concentrations. Specifically, we show how this matrix can be calculated from the transfer entropy between nodes, which is a measure of the flow of information between nodes and thus indirectly of the reaction propensity between them. We implemented a procedure of spectral graph embedding to embed the distance/dissimilarity matrix in flat and curved metric spaces, and consequently to determine the optimal latent geometry of the network. The distances between nodes in the metric space describing the latent geometry can be analyzed to identify bottlenecks in the reaction system. As a case study for this procedure, we consider the pharmacokinetics of atorvastatin, as described by recent studies and experimental time data.

**Conclusions:**

The method of determining distances between nodes from temporal measurements of node concentrations through the calculation of transfer entropy makes it possible to incorporate the information of kinetics (inherent in the time series) in the construction of the distance/dissimilarity matrix, and, consequently, in the determination of the network latent geometry, a characterisation of the network itself that is intimately connected to its dynamics, but which has so far been scarcely investigated and taken into account. The results on the case study of the pharmacokinetics of atorvastatin corroborate the usability and reliability of the method within certain limits of the experimental errors on the data.

## Introduction

A popular way in data science to describe a graph is the *dissimilarity matrix*. Its entries are the pairwise distinctions between the nodes of the graph. The dissimilarity matrix is a square $N \times N$ matrix (where $N$ is the number of nodes) with the $ij$-th element equal to the value of a chosen measure of distinction between the node $i$ and the node $j$. The measure of distinction is context specific, since it depends on the network under study (e.g. biological, social, IT, or market network), and the interactions between nodes which we want to focus on (e.g. chemical affinity in biochemical networks, co-expression in gene networks, statistical correlation, etc.). A special type of dissimilarity matrix is the distance matrix, whose entries are the distances between the nodes in a metric space.

**Fig. 1:**
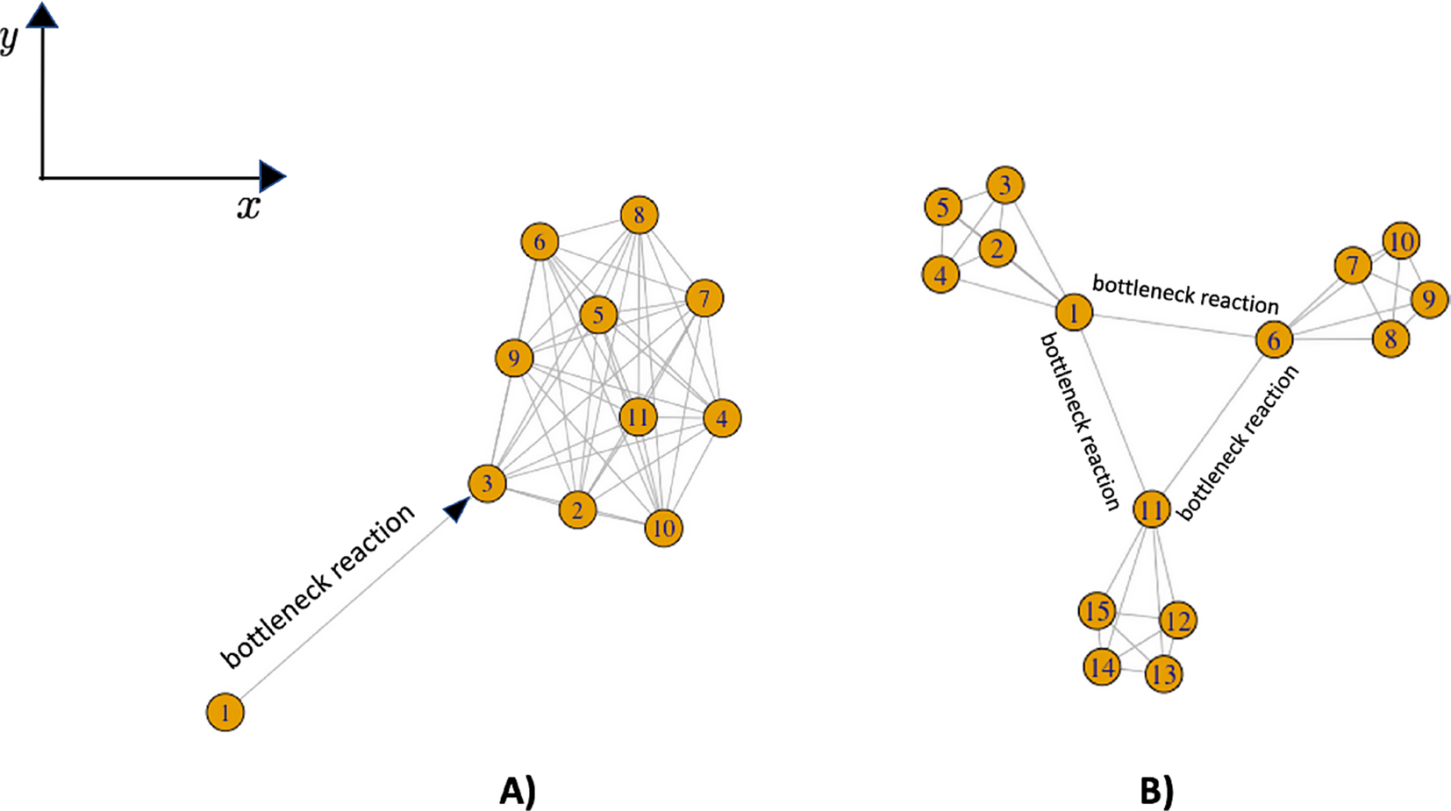
The spatial distance of the nodes located in the metric space describing the latent geometry of the network can be thought of as a measure of the propensity of interaction between in nodes, so that interactions between neighbouring nodes are characterised by a higher propensity than interactions between nodes that are far apart. When, as in (**A**), a node interacts with one or more nodes of a cluster located at a great distance from it and results in the staggered activation of interactions between the nodes of the cluster, the arc(s) connecting this node to the nodes of the cluster may identify bottlenecks for the chemical reaction system. In fact, the occurrence of reactions between the cluster nodes depends on the activity of the low-propensity interaction between the source node and the cluster nodes located at a great distance from it. Reactions that can be assumed to be bottlenecks for network dynamics can be those due to interactions between network modules as shown in (**B**)

The dissimilarity matrix is the simplest form in which a graph can be handled with computational procedures and therefore the knowledge of it is of considerable importance. It is not always possible to know this matrix as direct experimental data; thus the development and application of computational methods that calculate the matrix from the experimental data that can most commonly and easily be collected are necessary. The dissimilarity/distance matrix of a graph contains important information about the structure of the graph and the presence of any bottlenecks in the system of interactions described by the arcs. The objectives of this study are precisely to propose a method for calculating the distance matrix of a graph from the time series describing the dynamics of its nodes and, to identify of possible bottlenecks in the dynamics of the graph by embedding the distance matrix in a metric space. Our solution is to derive the dissimilarity between interacting nodes by using the transfer entropy. In the interaction between a node $X$ and a node $Y$, the transfer entropy from $X$ to $Y$ is the amount of information that node $X$ transmits to node $Y$ and which causes the variability of the quantitative features related to node $Y$ (e.g. concentration, reaction propensity) [1]. The embedding of the dissimilarity matrix in a space makes it possible to find the position coordinates of the nodes in that space and to interpret dissimilarity as the distance between nodes defined by the metric of the space. The analysis of the distances between nodes in the metric space that most accurately represents the latent geometry of the network allows the identification of bottlenecks in the dynamics of the interactions of the network nodes. Here by “bottlenecks” we mean interactions between nodes located at large distances, which by virtue of this take longer or have to overcome significant energy thresholds to take place. For example, clusters of nodes placed at a short distance from each other in the metric space describing the network’s geometry and activated by nodes placed at a great distance from them define a structure that could highlight possible bottlenecks in the kinetics and dynamics (see Fig. 1).

We illustrate our method on a biochemical network representing the pharmacokinetics of atorvastatin, a medication to reduce blood levels of lipids called triglycerides and cholesterol. This medication may help avoid health issues (such as heart attacks, strokes, and chest pain) brought on by fats obstructing blood vessels. Indeed, cardiovascular diseases remain the leading cause of death globally despite the current standard of care. Atherosclerosis, along with its clinical manifestations, such as myocardial infarction and ischaemic stroke, leads to a major burden on life expectancy, quality of life, and societal costs [2]. Dyslipidaemia is a major risk factor for atherosclerotic cardiovascular disease, with one-third of ischaemic heart disease being attributable to high cholesterol.

The theoretical study we propose in this article is intended as a contribution to the analysis of bottlenecks. Bottleneck analysis is indeed a useful technique for detecting inefficiencies and streamlining procedures in drug pharmacokinetics analysis and design. Concentrating on the parts of a pharmacokinetic network that experience bottlenecks can help increase drug efficiency, potentially minimise side effects, and ultimately improve the medicine’s quality. The identification and analysis of bottlenecks in biological networks, although recognised as being of fundamental importance, is still in its infancy. To the best of our knowledge, the identification of bottlenecks is carried out on the basis of centrality measurements, a priori knowledge of the kinetics or dynamics of the network (e.g. on the kinetic parameters of reactions, or on chemical binding affinities, or on statistical measures such as the correlation between nodes), dynamic sensitivity analysis, and phase space analysis, as we can find in [3–12]. The latent geometry of the network is not taken into account for the characterisation of interactions such as bottlenecks. The innovative contribution of this study is precisely the characterisation of an interaction as a bottleneck through the distance of the participating nodes in the network metric space, which is the structure that, unlike standard centrality measures, contains the laws of evolution of a network over time.

Precisely because the metric space and the dynamics of a network are intimately connected [13, 14], as the former determines the latter, which in turn can modify the structure of the former, in this study we derive the latent metric from the time series of the nodes in the network. From the time series we calculate the transfer entropy, which we interpret as a measure of the information volume related to the interaction propensity of the nodes. From this volume we calculate the distance between nodes, which is the input of graph embedding procedures for the identification of the network’s latent geometry. The bottlenecks, defined as interactions that are crucial for the functioning of the entire network, but which occur between nodes located at a significantly large distance, are thus identified through a procedure that takes into account the topology, the latent geometry of the network and the dynamic data expressed by the time series of the nodes.

The main advantage of the method we propose is that it does not implement iterative procedures, typical for example of sensitivity analysis, nor the exploration of the phase space, which is particularly complex if the data is affected by experimental error or non-negligible variances. Furthermore, our method does not require prior knowledge beyond experimental time series data.

The article is organized as follows: in Section “Related work” we report the main current literature relevant to the bottleneck identification in systems biology and bioinformatics; in Section “Atorvastatin pharmacokinetics” we present atorvastatin, its pharmacokinetics and recent literature supporting the current knowledge of the mechanisms of action and metabolism of the drug. In the Section we also present the experimental data that we use in our study (Sub-section “Data”). In Section “Methods” we introduce the concepts and definitions of the transfer entropy theory and its use for the calculation of distances between nodes of the atorvastatin network. In the same section we give brief hints on the embedding of a graph in flat and curved metric spaces. In Section “Results” we present the results of the study, i.e. the identification of the network’s latent geometry and the identification of bottlenecks interactions, and in Section “Discussion”, we comment on the results. Finally, Sections “Conclusions” is devoted to the concluding remarks, and “Appendix: Network latent geometry” gives some hints and literature references on the embedding of graphs.

## Related work

Besides the pharmacokinetics-oriented case study here employed, bottleneck identification is useful in systems-level study of cellular information processing [15], as well as in industrial biotechnology for production of chemicals, enzymes, antibiotics, and healthcare products [16]. Many historical attempts to identify bottlenecks have been at best semi-empirical. However, given the development of genetic and protein engineering tools, the question arises as to how one might rationally seek to identify the most promising gene or gene products to modify for the purpose of interest [17, 18].

An established approach adopted in bottleneck detection relies on sensitivity analysis, either under steady state conditions or under dynamic conditions, often in combination with models of metabolic pathways [9, 19]. The former approach is usually carried out by methods such as biochemical systems theory [20] and metabolic control analysis [21–23], whereas dynamic sensitivity analysis [24] by methods such as the Green’s function matrix analysis [25] and the impulse parametric sensitivity analysis [26] and its extension to account for pathway-level perturbations in dynamical pathway-based parametric sensitivity analysis [27].

The study of connectivity patterns in networks through edge centralities [28, 29] such as edge betweenness [30], edge closeness, edge eigenvector centrality, and nearest-neighbour edge centrality [31, 32] are deemed useful indicators for the identification of bottlenecks in network models and in real-world networks [33]. Furthermore, several statistical [34–37] and machine learning [38, 39] models have been developed, with the objective of identifying bottleneck locations.

Finally, there exist extensive studies in various disciplines, e.g. urban planning, traffic complexity, sustainable production system management [40], or routing in computer networks, focusing on bottlenecks identification and their spatio-temporal dynamics [41, 42] that could be borrowed in life sciences. Some of them explored the occurrences of congestion, including the kinematic wave theory [43, 44], the cellular automaton models [45, 46], and the three-phase traffic theory [47]. Attention has also been paid to understanding the bottleneck formation for the known causes, including the queue model [48], the lane-changing model [49], and the cell transmission model [50].

## Atorvastatin pharmacokinetics

Hydroxymethylglutaryl-coenzyme A (HMG-CoA) reductase inhibitors, commonly known as statins, are cornerstone of drug therapy for atherosclerotic cardiovascular disease. Most of the benefits of statin therapy are due to the lowering of serum total cholesterol levels, with the level of low-density lipoprotein cholesterol decreased and the level of high-density lipoprotein cholesterol increased [51]. Statins reduce the risk of major vascular events such as coronary deaths or myocardial infarctions, strokes in patients with known atherosclerotic cardiovascular disease [52–54] as well as in patients who are at increased risk but have not yet manifested a vascular event [55, 56]. Although generally smoothly tolerated by the organism, statins are associated with adverse drug reactions in a small subset of patients, including statin-related myotoxicity [57–60]. The clinical spectrum of statin-induced myotoxicity varies greatly from asymptomatic elevations of creatine kinase (CK) without muscle pain, to muscle pain or weakness with raised CK levels, myositis with biopsy-proven muscle inflammation, and, finally, rhabdomyolysis with muscle symptoms, high CK, and potential for acute kidney injury [61, 62]. Risk factors include higher statin dose, comedications, and potentially increased circulating levels of statin lactone species, which are considered more myotoxic, as well as genetic factors [63, 64].

Among statins, atorvastatin (AS) is the guideline-recommended first-line lipid-lowering drug. Atorvastatin is administered orally as a calcium salt in the active acid form with a clinical dosage ranging commonly from 10 to 80 mg/day. AS is rapidly absorbed, reaching peak plasma concentration within 4 h in immediate-release formulations [65]. AS is transported systemically either through passive diffusion or actively assisted by endogenous carriers such as members of the organic anion-transporting polypeptide (OATP) family [66, 67]. Bio-transformation of the pharmacologically active AS occurs in the liver. The liver-specific OATP family members [68] OATP1B1 [67, 69, 70], which is encoded by gene SLCO1B1, OATP2B1 [66, 71], which is encoded by gene SLCO2B1, and OATP1B3 [72] regulate the uptake of AS into hepatocytes, increasing the amount of drug available for metabolism by liver enzymes. The reduced AS hepatic uptake and the consequently reduced hepatic formation of its active metabolites can decrease their therapeutic efficacy and promote the onset of adverse reactions such as rhabdomyolysis or myopathy [73]. Less than 1% of atorvastatin and derivatives are eliminated in urine [74], which points at AS excretion mainly by hepatobiliary mechanisms. AS is actively exported out of the hepatocytes into the bile by the ATP-dependent multidrug resistance gene 1 (MDR1, ABCB1) transporter [75, 76], and by the multidrug resistance-associated protein 2 (MRP2, ABCC2) [70].

Active AS is transformed to its corresponding inactive lactone form (ASL) by different UDP-glucuronosyltransferase (UGT) enzymes, the most important of which is UGT1A3 [77, 78]. Both AS and ASL are further metabolized into their para- and ortho-hydroxy-metabolites, ASpOH, ASoOH, ASLpOH and ASLoOH, by cytochrome P450 (CYP) enzymes, mainly CYP3A4 and CYP3A5, and, to a lower extent, CYP2C8 [79–81]. The main metabolite, 2-hydroxy-atorvastatin, is pharmacologically active and significantly contributes to the inhibitory activity on HMG-CoA reductase. The lactone forms of atorvastatin and its metabolites can also be hydrolyzed back into their corresponding acid forms either non-enzymatically or by paraoxonases [82–84]. Genetic polymorphisms in the genes coding for these proteins involved in the absorption, distribution, metabolism, and excretion processes have been extensively investigated [67, 85–87], mainly through association studies using non-compartmental pharmacokinetic analysis on healthy volunteers after single dose administration [63, 88–90].

Quantitative structure-activity relationship (QSAR) [91, 92], that is a computational modelling method for unveiling the relationships between structural properties of chemical compounds and their physicochemical and biological properties, is widely used in computer-aided drug design. A combination of molecular modelling techniques including three-dimensional quantitative structure-activity relationship (3D-QSAR), molecular docking and molecular dynamics simulation was employed to explore the feasibility of atorvastatin analogues as HMG-CoA reductase inhibitors [93].

Considerable progress has been made towards predicting pharmacokinetic behaviour from *in vitro* information on the interaction between atorvastatin and enzymes and atorvastatin and additional compounds [94, 95]. However, data on *in vitro* human drug metabolism could be deemed in lack of appropriate depth for instructing the implementation of in vivo studies in humans. Furthermore, in vitro studies are limited in the fact that they do not account for inter-subject variability.

The informative value of the data routinely generated during *in vitro* atorvastatin studies on its physicochemical properties such as permeability, solubility, lipophilicity [96, 97] and biological properties such as receptor binding, and metabolic stability [98, 99] is usually exploited in mechanistic and physiologically based pharmacokinetic (PBPK) models in the context of simulations and predictions of absorption, distribution, metabolism and excretion processes in virtual patient populations [100]. PBPK models integrate experimentally based information and mechanistic framework of physiological and biological processes using implicit and explicit assumptions by relying on drug-, system- and trial design-related parameters. PBPK models are positioned as a valuable tool for the characterization of complex pharmacokinetic/pharmacodynamics (PK/PD) processes and its extrapolation in special sub-groups of the population [100]. Several PBPK models of AS have been published in the recent years [97], which have been often carried out within the Simcyp PBPK simulator [101]. PBPK models address different aspects of the PK/PD properties of AS such as dose selection, exploration of drug-drug interactions. PBPK models have been applied to quantitatively predict drug-drug interaction (DDI) effects. For instance, the PBPK model for atorvastatin and its two hydroxy-metabolites, 2-hydroxy-atorvastatin acid and atorvastatin lactone [102], aimed at predicting the pharmacokinetic profiles and DDI effects by examining different DDI scenarios, where atorvastatin was coadministered with a CYP3A4 inhibitor (itraconazole, clarithromycin, or cimetidine), or CYP3A4 inducer (rifampin or phenytoin). The model developed in [103] integrated the model introduced in [102] by accounting for the active uptake mediated by OATP1B3 and for the biliary excretion of AS. Another atorvastatin PBPK model was developed using *in vitro* and human pharmacokinetic data by considering the contribution of both metabolizing enzymes and transporters to the disposition of the drug [104]. The PBPK model was used to simulate statin pharmacokinetic in subjects with varying SLCO1B1 polymorphism or in subjects co-administered with various CYP enzymes and/or transporter inhibitors. As shown in the previously mentioned PBPK models, AS disposition is determined by cytochrome P450 (CYP) 3A4 and polypeptides (OATPs). Since drugs that affect gastric emptying, including dulaglutide, affect atorvastatin pharmacokinetics, a recent PBPK model sought to include gastric acid-mediated lactone equilibration of atorvastatin to predict atorvastatin acid, lactone, and their major metabolites [105]. More recently, a PBPK model for atorvastatin and its metabolites was developed to predict their pharmacokinetics upon administration of solid oral dosage of AS calcium salt at several dosage levels in single and multiple dosing schedules [106]. Differently from previous models, this model accounts also for AS solubility-limited absorption in the attempt to improve clinical trial design and real-life administration schedules.

### Data

**Fig. 2:**
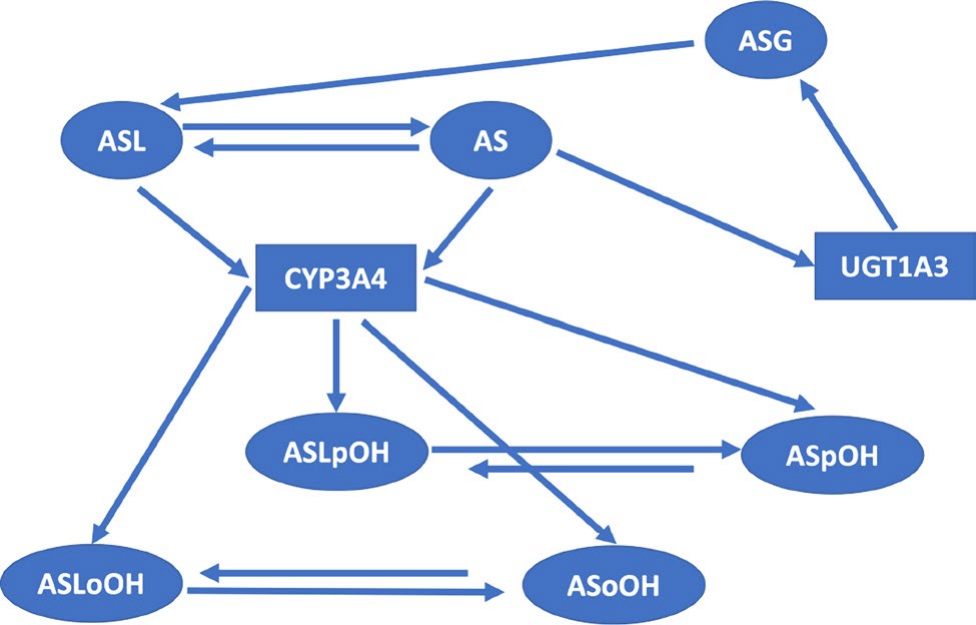
Simplified scheme of the intracellular model of atorvastatin metabolism in primary human hepatocytes [107]. The model includes AS and ASL, and their para- and ortho-hydroxy-metabolites, ASpOH and ASoOH, ASLpOH and ASLoOH. AS and ASL are hydroxylated to the corresponding metabolites by CYP3A4. Compound AS is converted via an unstable glucuronide-intermediate (ASG) to ASL mediated by UGT1A3

**Fig. 3:**
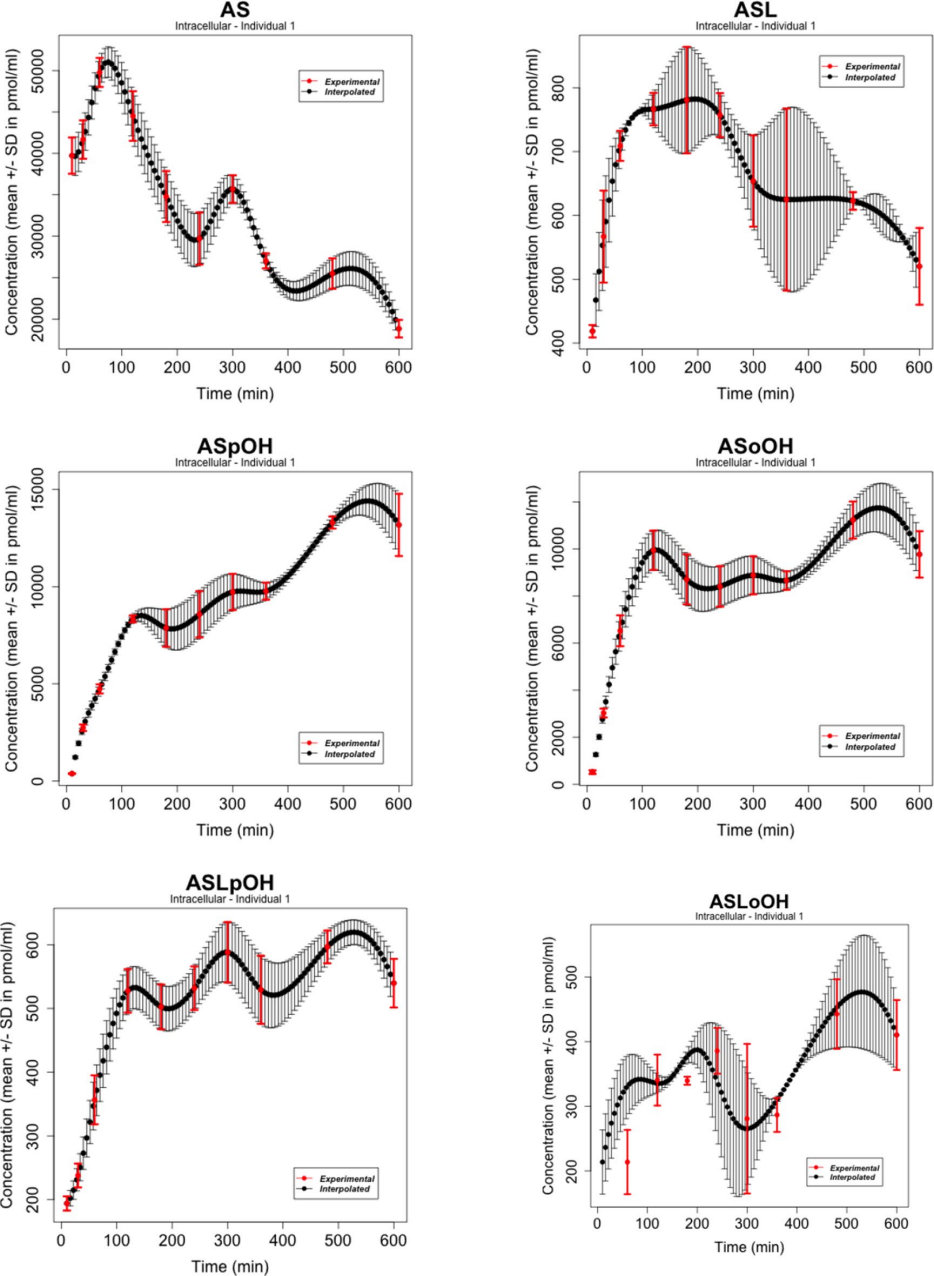
Atorvastatin metabolite intracellular concentrations in the time-series experiment on primary human hepatocytes of Individual 1 from LC-MS/MS measurements as in [107]. The experimental points (in red) as well as the width of the error bars have been interpolated with the spline FMM method [108]

**Fig. 4:**
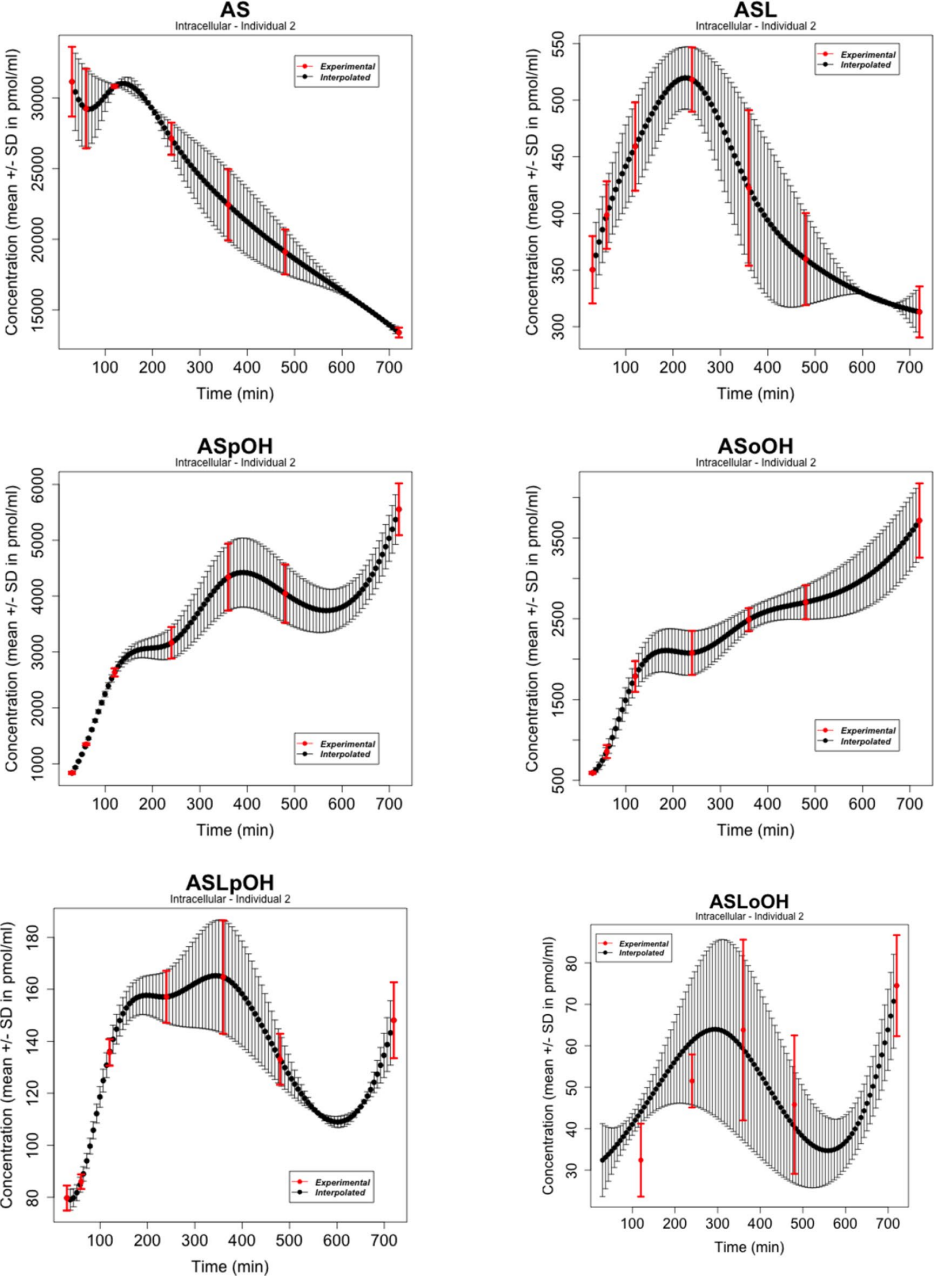
Atorvastatin metabolite intracellular concentrations in the time-series experiment on primary human hepatocytes of Individual 2 from LC-MS/MS measurements as in [107]. The experimental points (in red) as well as the width of the error bars have been interpolated with the spline FMM method [108]

**Fig. 5:**
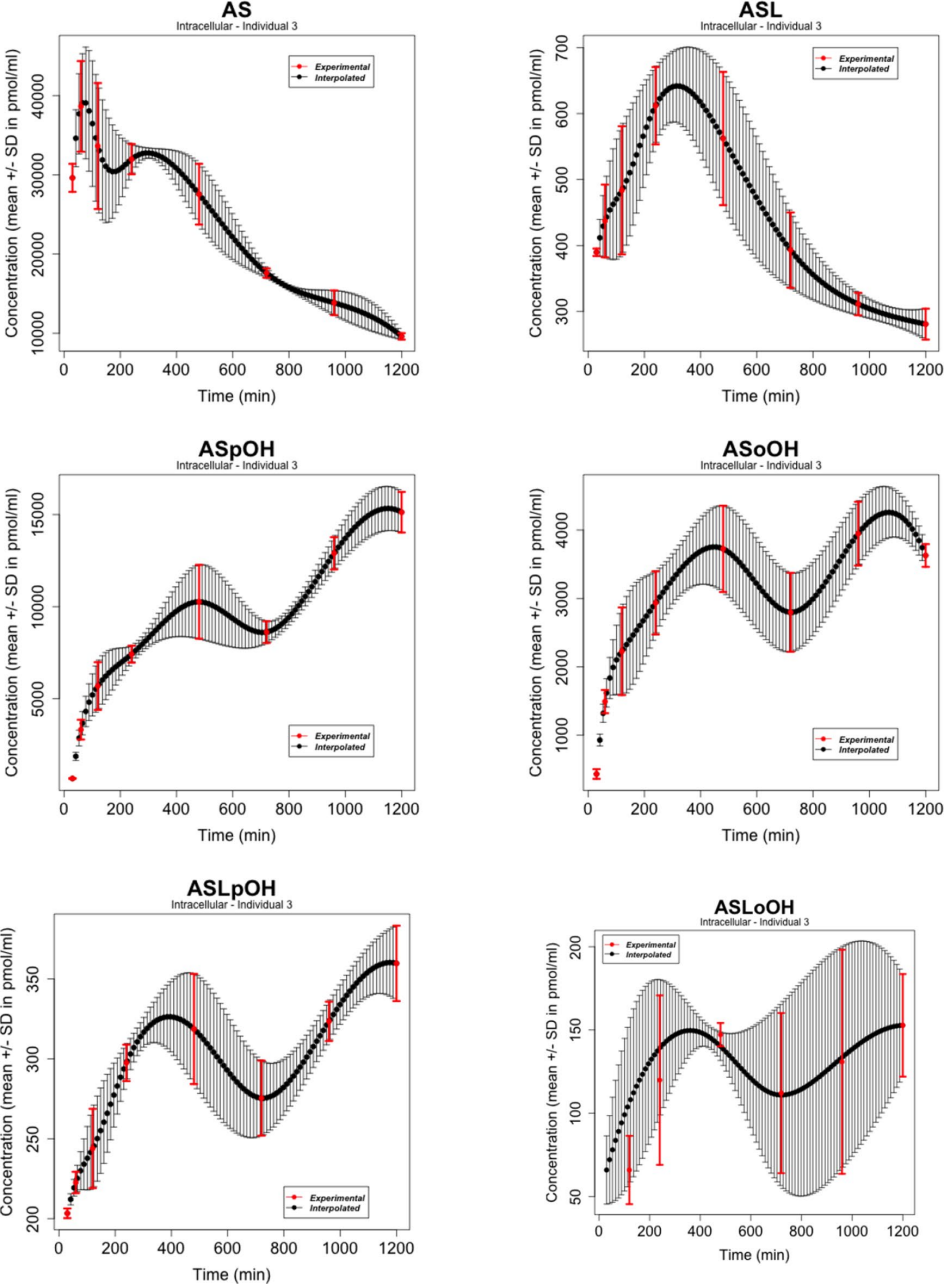
Atorvastatin metabolite intracellular concentrations in the time-series experiment on primary human hepatocytes of Individual 3 from LC-MS/MS measurements as in [107]. The experimental points (in red) as well as the width of the error bars have been interpolated with the spline FMM method [108]

We considered atorvastatin metabolite concentrations in the time-series experiment on primary human hepatocytes of three individuals as reported by Bucher et al. in [107], where atorvastatin acid and lactone (AS and ASL) and corresponding para- and ortho-hydroxy-metabolites (acids: ASpOH and ASoOH; lactones: ASLpOH and ASLoOH) have been measured at the defined time points with mean and standard deviation ($n = 3$) from measurements per liquid chromatography/mass spectrometry (LC-MS/MS). The data refer to the nodes of the simplified scheme of interactions in Figure 2. Figures 3, 4, 5 show the behaviours of the time series in [107] whose number of points was augmented with a cubic spline interpolation method of Forsythe, Malcolm, and Moler (FMM) [108]. The spline interpolation was applied to both the experimental measurements and the magnitude of the error bars, given by an average value over three measurements per individual and the standard deviation, respectively. Cubic splines traverse all the data points, ensure a certain level of precision, mitigate - to a certain degree - the amplitude of fluctuations due to stochastic effects and/or experimental uncertainties, and provide smooth functions. Specifically, cubic splines are continuous from the zeroth to the second derivative. This is a property required for a time curve to describe kinetics. The interpolation obtained here contains all the experimental time points. The time series data refer to the nodes of the simplified scheme of interaction in Fig. 2.

The computational procedure proposed in this study uses the graph of the interaction network and the time series of its components as input data. The procedure can be applied to both direct and indirect graphs (and multigraphs).

## Methods

We first briefly introduce some fundamental quantities of the information theory related to the concept of transfer entropy useful for understanding the steps of the application we make in this study. The reader can find in [109-116] a more comprehensive review of modern approaches of information theory in various applicative domains. We then present the procedure for deriving distances between nodes from the transfer entropy, and a short summary of the graph embedding for the identification of latent geometry.

### Transfer entropy

Consider a random variable $X$ drawn from a sample space ${S_X}$. The amount of information associated with the event $X = x$ is

1 $$I(X = x) = - \mathop {\log }\nolimits_2 \Pr (X = x).$$

The random variables X and Y might be either independent or dependent on one another. One random variable includes information about another in the situation of dependency. The mutual information $I(X,Y)$ quantifies the amount of information that $X$ has about $Y$ (or vice versa)

2 $$\eqalign{ I(X(t),Y(t)) = & \sum\limits_{\scriptstyle {x_t} \in {S_X}, \atop \scriptstyle {y_t} \in {S_Y}} {\Pr } (X(t) = {x_t},Y(t) = {y_t}) \cr & {\log _2}{{\Pr (X(t) = {x_t},Y(t) = {y_t})} \over {\Pr (X(t) = {x_t})\Pr (Y(t) = {y_t})}}. \cr} $$

Since, according to Equation (2), the mutual information is symmetric, i.e., $I(X,Y) = I(Y,X)$, it cannot be used for causal inference. If the present of one variable (effect) is determined by the past of another variable (cause), the causal direction (direction of information flow) from the cause to the effect can be inferred. By introducing a time-lag parameter $\tau$ in any of the variables $X$ and $Y$, one can create an asymmetric measure known as time-delayed mutual information, that is

3 $$\eqalign{ I(X(t + \tau ),Y(t)) = & \sum\limits_{\scriptstyle {x_t},\;{x_{t + \tau }} \in {S_X}, \hfill \atop \scriptstyle {y_t} \in {S_Y} \hfill} {\Pr (X(t + \tau ) = {x_{t + \tau }},Y(t) = {y_t}) \times } \cr & \times {\log _2}{{\Pr (X(t + \tau ) = {x_{t + \tau }},Y(t) = {y_t})} \over {\Pr (X(t + \tau ) = {x_{t + \tau }})\Pr (Y(t) = {y_t})}} \cr} $$

The joint entropy and the conditional entropy for two random variables $X$ an $Y$, drawn from sample spaces ${S_X}$ and ${S_Y}$ respectively, are

4 $$\eqalign{ H(X(t),Y(t)) = - \sum\limits_{\scriptstyle {x_t} \in {S_X}, \atop \scriptstyle {y_t} \in {S_Y}} {\Pr } & (X(t) = {x_t},Y(t) = {y_t}){\log _2} \cr \Pr & (X(t) = {x_t},Y(t) = {y_t}) \cr} $$

and

5 $$\eqalign{ H(X(t)|Y(t)) = - \sum\limits_{\scriptstyle {x_t} \in {S_X}, \atop \scriptstyle {y_t} \in {S_Y}} {\Pr } & (X(t) = {x_t},Y(t) = {y_t}){\log _2} \cr \Pr & (X(t) = {x_t}|Y(t) = {y_t}). \cr} $$

The transfer entropy (TE) from $Y$ to $X$ is the difference between the entropy of $X(t + \tau )$ conditioned on $X(t)$ and that conditioned on both $X(t)$ and $Y(t)$ [117], i.e. the TE from $Y$ to $X$ is

$$\begin{aligned} {\text{T}}{{\text{E}}_{Y \to X}} & = I(X(t + \tau ) = {x_{t + \tau }},Y(t) = {y_t}|X(t) = {x_t}) \\ & \, = H(X(t + \tau )|X(t)) - H(X(t + \tau )|X(t),Y(t)) \\ \end{aligned} $$

that is

6 $${\rm{T}}{{\rm{E}}_{Y \to X}} = \left\{ {\sum\limits_{\scriptstyle {x_{t + \tau }},{x_t} \in {S_X}, \atop \scriptstyle {y_t} \in {S_Y}} C ({X_t},{x_{t + \tau }},{y_t})\;{{\log }_2}{\eqalign{\Pr (X(t + \tau ) & = {x_{t + \tau }}|X(t) \cr & = {x_t},Y(t) = {y_t}) \cr} \over \matrix{\Pr [X(t + \tau ) \hfill \cr = {x_{t + \tau }}|X(t) = {x_t}] \hfill \cr} }} \right\}.$$

where $$\tau $$ is a time lag, $$C({x_t},{x_{t + \tau }},{y_t}) \equiv \Pr (X(t + \tau )$$$$= {x_{t + \tau }},X(t) = {x_t},Y(t) = {y_t})$$. TE quantifies the reduction in uncertainty associated with predicting $$X(t + \tau )$$ from both $$X(t)$$ and $$Y(t)$$ in comparison to predicting it from $$X(t)$$ alone. A positive $${\text{T}}{{\text{E}}_{Y \to X}}$$ suggests that the past of $$Y$$ provides some knowledge about $${x_{t + \tau }}$$ that the past of $$X$$ does not, indicating that $$Y$$ has a causal influence on $$X$$. Because a follower agent follows the motion of a leader but not the other way around, a leader may more precisely predict the motion of a follower. A follower, on the other hand, cannot forecast a leader with such accuracy. Usually, net TE from $$Y$$ to $$X$$ is considered as $${\text{NT}}{{\text{E}}_{Y \to X}} = {\text{T}}{{\text{E}}_{Y \to X}} - {\text{T}}{{\text{E}}_{X \to Y}}$$. As a result, a positive $${\text{NT}}{{\text{E}}_{Y \to X}}$$ shows that $$X$$ follows $$Y$$.

### Transfer entropy as volume of information

**Fig. 6:**
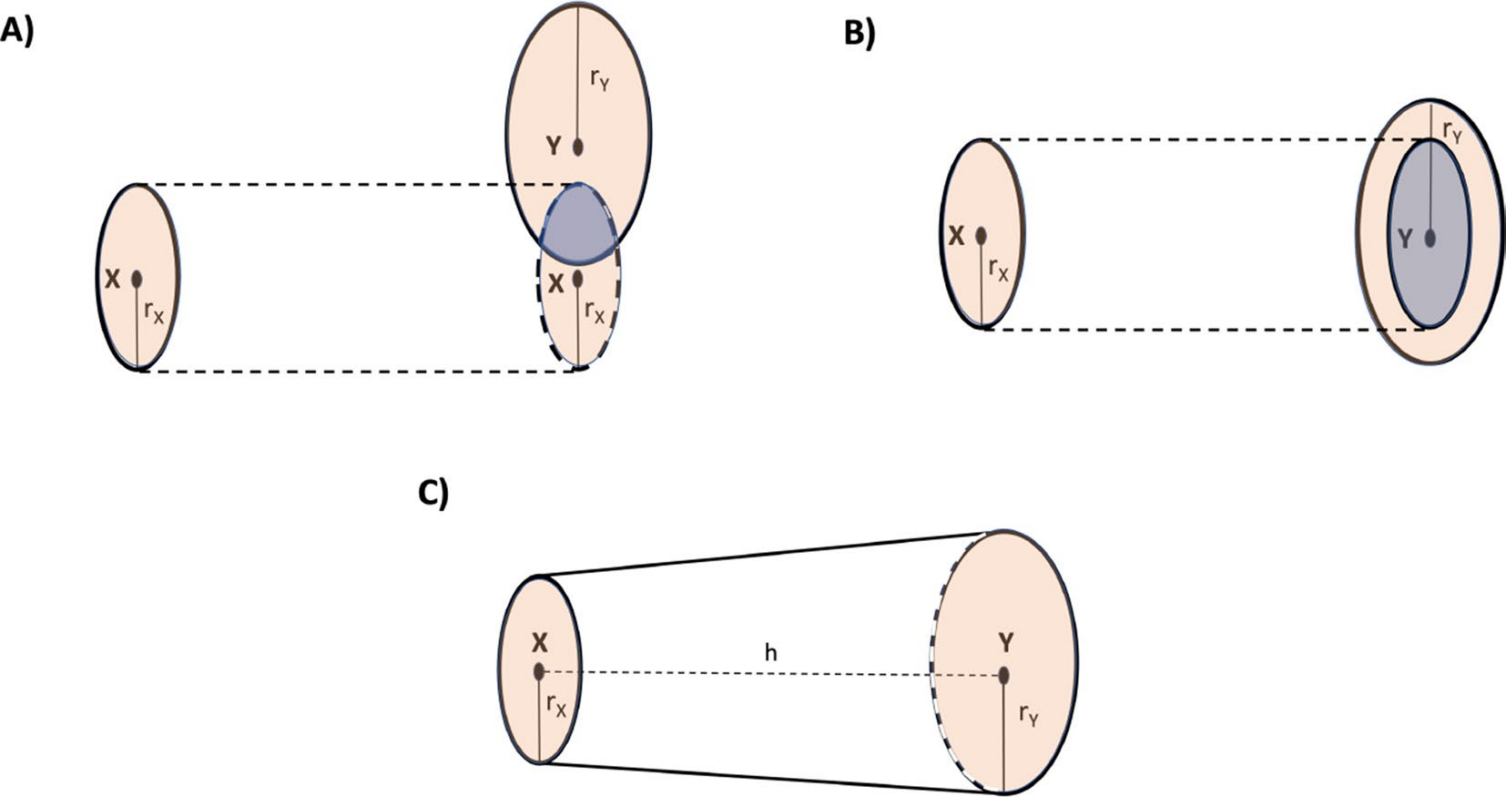
The area of an imaginary circle centred in particle $X$ with radius ${r_X}$ quantifies the reaction propensity of particle $X$, i.e. the amount of information the particle is able to transmit to particle $Y$. A similar definition is given for the area of the circle centred in $Y$ and having radius ${r_Y}$. The interaction between the particles is successful if the orthogonal projection of one circle onto the other has a maximum area. In (**A**) the case is shown where the orthogonal projection of the circle of $X$ onto $Y$ (grey shaded area)has no maximum area, equal to the area of the circle of $X$. In (**B**), on the other hand, the case is shown where the circles centred in the two parcels are arranged parallel to each other so that the area of the orthogonal projection of the circle of $X$ onto the circle of $Y$ is maximum. The configuration in (**B**) makes it possible to define a truncated cone volume as in figure (**C**) and to derive from it the distance h between the particles under the condition of maximum transmission efficiency of information between the two

Considering that two nodes cannot communicate across an indefinite distance, we define an optimal distance at which all information can be exchanged between two nodes $X$ and $Y$ in both directions (from $X$ to $Y$ and *vice versa*). To this purpose, we introduce a cross section, defined as the area measured around $X$ within which the presence of node $Y$ causes interaction phenomena between the two bodies. Let us assume, for simplicity’s sake, that the cross section is circular. We consider the interaction to be maximally probable and effective when the circles define the bases of a truncated cone. In this way, ideally in this representation, the maximum contact area for interaction is exposed (see Fig. 6A, B and C).

The height $h$ of the truncated cone quantifies the distance at which the interaction node $X$ and node $Y$ is maximally efficient. We set the volume $V$ of the truncated cone as in Eq. (7).

7 $$V = \left\{ {\begin{array}{*{20}{c}}{T{E_{X \to Y}}}&{{\text{if}}\,T{E_{X \to Y}} \ne 0\,{\text{and}}\,T{E_{Y \to X}} = 0} \\ {T{E_{Y \to X}}}&{{\text{if}}\,T{E_{Y \to X}} \ne 0\,{\text{and}}\,T{E_{X \to Y}} = 0} \\ {T{E_{X \to Y}} + T{E_{Y \to X}}}& {{\text{if}}\,T{E_{Y \to X}} \ne 0\,{\text{and}}\,T{E_{X \to Y}} \ne 0.} \end{array}} \right.$$

The null value of TE is statistically determined if the p-value is greater than 10%. We chose the highest significance threshold of those commonly used in a statistical test, since the data we used have a low sample size and are affected by a large variance. In this way we increase the sensitivity of the statistical test on the TE value.

In Box 1 we see, for example, the transfer entropy calculated by the R library function RTransferEntropy [110, 118] for the AS and ASL time series for Individual 1. We point out that in our model, in the case in which both the transfer entropies are different from zero the volume is defined as the sum of the transfer entropy, and not as the net transfer entropy, because our intention is precisely to calculate the volume as the size of the region containing both flows of information, i.e. what $X$ transmits to $Y$ and what $Y$ transmits to $X$.
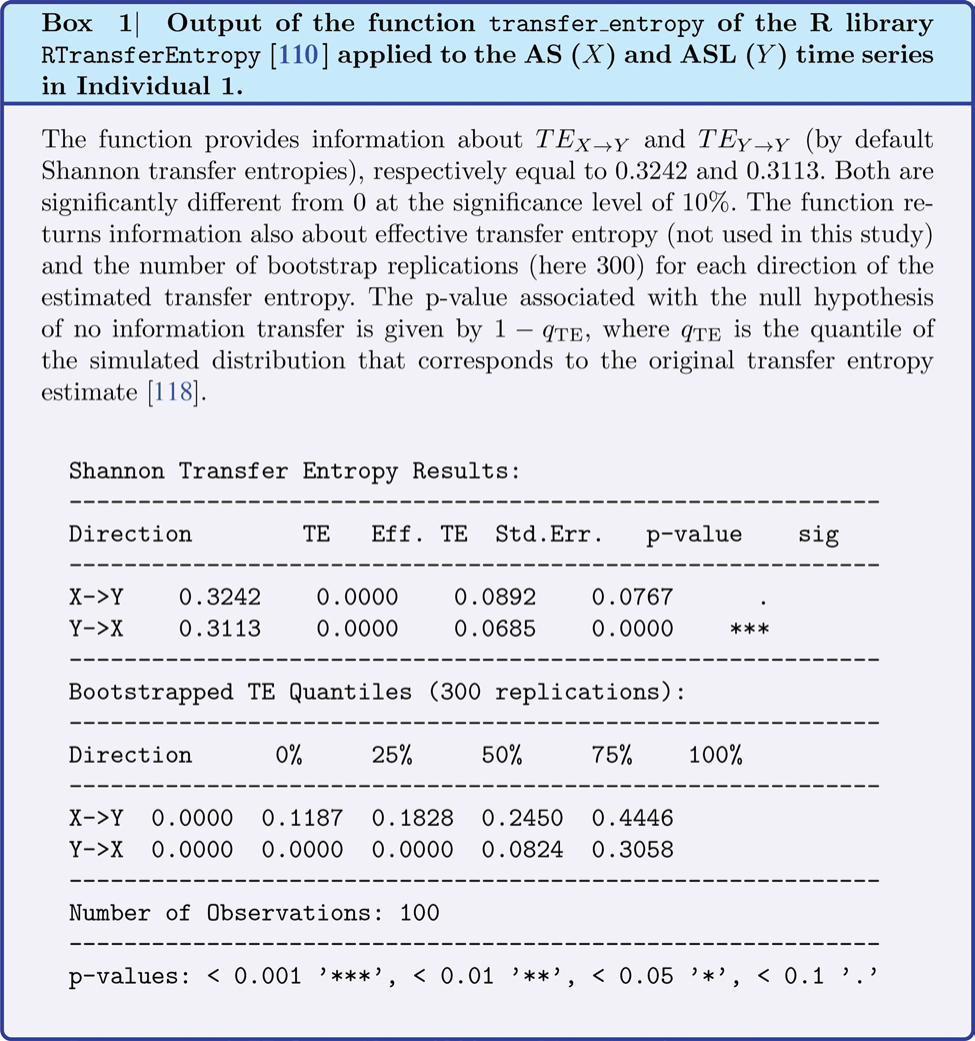


In the case in which both $T{E_{X \to Y}}$ and $T{E_{Y \to X}}$ are not significantly different from zero, there is not interaction between $X$ and $Y$. On the other hand, the volume of the truncated cone is given by

8 $$V = \frac{1}{3}\pi (r_X^2 + {r_X}{r_Y} + r_Y^2)h,$$

where $h$, ${r_X}$, and ${r_Y}$ are the height, and the radii of the bases as in in Fig. 6C. In this model, the areas of the circles of the truncated cone bases represent a measure of the nodes propensity to transmit their information, a concept that evokes the reaction propensity in the stochastic, molecular-level approaches of chemical kinetics [119]. Based on this representation, these area are estimated as time derivative of the transfer entropy since this quantity measures (and ranks) the rate of change in the flow of information and thus identifies the tendency of a node to be more or less promptly “communicative”.

Given the input time series of length $n$ for two species $X$ and $Y$, we then formalize the definition of ${r_X}$ and ${r_Y}$ as follows:

9 $${r_X} = \frac{1}{n}\sum\limits_{i = 1}^n {\left| {\frac{\partial }{{\partial t}}{\text{T}}{{\text{E}}_{X \to Y}}(t)} \right|_{t = {t_i}}}\quad {\text{and}}\quad {r_Y} = \frac{1}{n}\sum\limits_{i = 1}^n {\left| {\frac{\partial }{{\partial t}}{\text{T}}{{\text{E}}_{Y \to X}}(t)} \right|_{t = {t_i}}}.$$

It should be noted that the formulae defining entropy transfer do not make it a function of time. However, by breaking the time-series arrays of chemical species concentrations into consequential subarrays over time, it is possible to calculate the amount of transfer entropy transmitted in the various time chunks relative to the subarrays. In this study we partitioned the 100 time point in chunks of 10 points.

Formula (9) returns a non-zero value if the time derivatives are different from 0. In the case in which the time derivative are null, ${r_X}$ and ${r_Y}$ are estimated as the absolute value of the angular coefficients ${m_X}$ and ${m_Y}$, of the straight lines fitting $X(t)$ vs $t$ and $Y(t)$ vs $t$, respectively, i.e.

10 $$\eqalign{ X(t) & = {m_X}t + {q_X},\quad {\rm{and}}\quad Y(t) \cr & = {m_Y}t + {q_Y},\quad {r_X} = \left| {{m_X}} \right|,{r_Y} = \left| {{m_Y}} \right|. \cr} $$

In fact, the time derivatives of the transfer entropy are zero when the two time series corresponding to the interacting nodes have a linear course in time. Therefore, in these cases, a measure of the reaction propensity is given through the rate of change of the time curve itself. Having the transfer entropy and radii, we derive $h$ from the Eq. (8) as

11 $$h = \frac{{3V}}{{\pi (r_X^2 + {r_X}{r_Y} + r_Y^2)}}.$$

Since the experimental points show a non-negligible variation from the range of variation of the time series, we considered it appropriate to estimate a range of variation for $h$. To this end, for each time series we randomly sampled 100 time series curves belonging to the interval defined by the error bars. The variation intervals on $h$, ${r_X}$ and ${r_Y}$ were then obtained as the standard error (SE) on the average of 100 estimates of the transfer entropies obtained from the 100 time series. If $\Delta V$, $\Delta {r_X}$, and $\Delta {r_Y}$ are the variation interval obtained from these simulations, the variation interval of $h$ is given by:

12 $$\Delta h = \frac{{3\sqrt {{{(V\Delta {r_X})}^2}{{(2{r_X} + {r_Y})}^2} + {{(V\Delta {r_Y})}^2}{{(2{r_Y} + {r_X})}^2} + {{(\Delta V)}^2}} }}{{\pi {{(r_X^2 + {r_X}{r_Y} + r_Y^2)}^2}}}.$$

The values of $h$ for each couple of nodes, ${h_{ij}}$, are arranged in the distance matrix $D = \{ {h_{ij}}$}.

### Graph embedding

In order to identify the geometry of the network defined by the distance matrix $D$, we embed the network in three metric spaces: Euclidean, hyperbolic and spherical and calculated the stress, i.e the distortion of the distance caused by the embedding.

The embedding stress is defined as:

13 $${\text{Stress}} = \frac{1}{\Xi }\sqrt {\sum\limits_{ij} {{({h_{ij}} - h_{ij}^ * )}^2}}.$$

where $$\Xi $$ is the number of nodes, $$h_{ij}^ * $$ is node distance as in the space in which the graph has been embedded.

The embedding with the smallest stress value determines the optimal latent geometry for the network.

In this study, we use the embedding method developed by P. Lecca, and P. Lecca et al. whose theoretical foundations, implementation details, and use cases can be found in [14, 120-122]. We give here a brief summary of the embedding method.

#### Embedding in Euclidean space

The matrix $U = [{u_1}{u_2} \ldots {u_m}]$ of the graph Laplacian eigenvectors provides the embedding in a Euclidean space of dimension $m$ ($U$ is a $m \times m$ matrix). According to increasing values of the respective eigenvalues, the eigenvectors are arranged in ascending order in a matrix, whose i*-th* row defines the coordinates of the node ${v_i}$ in Euclidean space.

#### Embedding in constant curvature manifolds

The spectral decomposition of the matrix

14 $$C_{ij}^{U,k} = \cos (\sqrt k {d_{ij}}).$$

where $\mathbb{U}$ is a space of dimension $n$, $d$ is a function such that $d:U \times U \to {\mathbb{R}^ + }$, $D = \{ {d_{ij}}\} = \{ d({u_i},{u_j})\} \}$ denotes the node-to-node distance matrix, and $k$ the curvature of the space, is calculated.

To verify the possibility of an isometric embedding the theorem of Blumenthal [123] and Schoenberg [124] are used. This theorem states that if $k < 0$, the space defined by $U$ can be isometrically embedded in $\mathbb{H}_k^m$ if and only if the number of positive, negative and zero eigenvalues of ${C^{U,k}}$ is $1,p$ and $n - p - 1$, respectively, where $qp \leq m$. If $k < 0$, the space spanned by $U$ can be isometrically embedded in $\mathbb{H}_k^m$ if and only if the number of positive, negative and zero eigenvalues of ${C^{U,k}}$ is $p,0$, and, $n - p$, respectively, with $p \leq m + 1$. If the curvature of embedding space is negative, Begelfor et al. [125] calculate the coordinates of node $i$ as

15 $${v_i} = \frac{1}{{\sqrt {1 - {{\left\| {{w_i}} \right\|}^2}} }}{U_m}\sqrt { - {\Sigma _m}} $$

where ${\Sigma _m}$ is the diagonal matrix of the $m$ most negative eigenvalue of ${C^{U,k}}$ and ${({w_1}\;{w_2}\; \ldots \;{w_n})^T} = {U_m}\sqrt { - {\Sigma _m}}$.

If the curvature is positive, the i-$th$ coordinates are

16 $${v_i} = \frac{1}{{\left\| {{w_i}} \right\|}}U\sqrt \Sigma.$$

If the conditions for an isometric embedding are not satisfied, the $C$ eigenvalue decomposition and the selection of the dominating $m$ eigenvectors are used.

### Identification of bottlenecks

Once the metric space of the graph has been identified, measurements of h, ${r_X}$ and ${r_Y}$ are used to identify possible bottleneck interactions, according to the following criterion. In the metric space of the network, we characterise an interaction between $X$ and $Y$ as a possible bottleneck, if ${r_X}$ or ${r_Y}$ have “small” values and $h$ is “large”. Vice versa, we characterise the reaction between $X$ and $Y$ as high-propensity reactions if ${r_X}$ or ${r_Y}$ have “high” values and $h$ “small” values. In order to separate small values from large values, we used the Triangle method. This method is an automatic thresholding method based on the histogram of the variable whose thresholds we want to find. A threshold is calculated based on the intensity range and greatest peak. The approach was proposed by Zack et al. [126]. It creates a line connecting the histogram peak and the farthest end of the histogram. The threshold is the greatest distance between the line and the histogram. We chose this method because it proved to be the most accurate on our data compared to other histogram-based thresholding methods we tested such as, IJDefault, Huang, Huang2, Intermodes, IsoData, Li, Mean, MinErrorI, Minimum, Moments, Otsu, Percentile, MaxEntropy, RenyiEntropy, and Shanbhag [127].

A necessary and sufficient condition for an interaction between $X$ and $Y$ to be considered as a bottleneck is that the following three conditions are all satisfied:

- $${r_X} < r_X^{{\text{(threshold)}}}$$,
- $${r_Y} < r_Y^{{\text{(threshold)}}}$$,
- $${h^ * } < {h^{( *,threshold)}}$$.

where $r_X^{{\text{(threshold)}}}$, $r_X^{{\text{(threshold)}}}$, and ${h^{( *,threshold)}}$ are the threshold values for ${r_X}$ be ${r_Y}$, and ${h^ * }$ calculated using the Triangle method.

Embedding the graph in a metric space is relevant to the bottleneck identification procedure. Indeed, embedding returns not only the optimal latent geometry of a network - through the comparison of the embedding stresses in different metric spaces, but also the coordinates of the nodes in the metric space

Knowing the coordinates is important, especially for curved spaces, and in particular hyperbolic space. In Poincaré’s representation, for example, points/nodes that are located close to the edge are points at infinity, i.e. points that are very distant from points located in areas of the disc closer to the origin. By virtue of this distance, these nodes, when in communication with nodes closer to the origin, are reactants of candidate bottlenecks. An example of this situation will be shown on the case study under consideration in this article. Moreover, in curved spaces, the distance is the length of a geodesic on which the points lie. The curvature of this geodesic is another parameter that can characterise an interaction as a bottleneck (e.g. strongly curved segments connecting interacting nodes/points could be indicative of bottleneck reactions).

### Summary of the computational pipeline

**Fig. 7:**
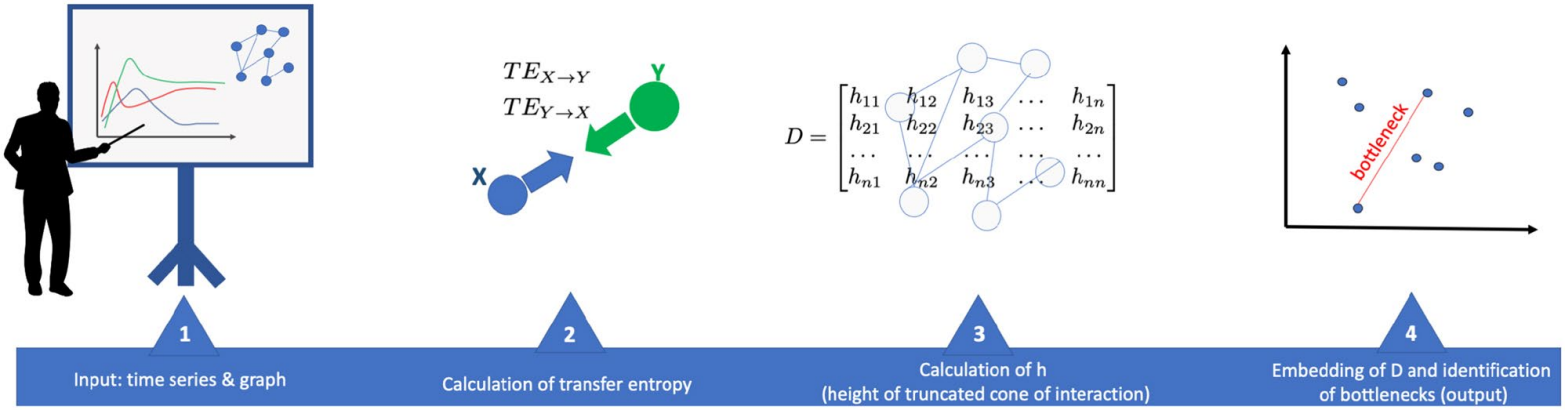
Steps in the computational procedure that from the time series of the system’s components and the graph of interactions derives the distance between molecules and then perform embedding in a metric space and identify, in this metric space, interactions that may be bottlenecks

In Fig. 7 we summarise the steps of the method: the input is the time series and the graph. From the time series we calculate the transfer entropy for each interaction on the graph shown in Fig. 2. From the transfer entropy thought of as the volume of a truncated cone quantifying the information exchanged between two interacting molecules, we calculate the distance between the two molecules. Finally, we embed the distance matrix thus obtained in the three metric spaces Euclidean, hyperbolic, and spherical. In the metric space representing the latent geometry of the network, we identify the bottlenecks.

## Results

Tables 1, 2, and 3 show, for Individuals 1, 2 and 3 respectively, the values of volume (transfer entropy) and interaction radii with their standard error. Tables 4, 5, and 6 show the distance between chemical species for which the interaction volume results other than zero. We observe that the presence of a considerable amount of variation in the experimental data of the time series causes a high standard error on the estimate of the distance $h$ (cases indicated as “high SE” in the tables). Table 7 shows the embedding stresses in 2D, indicating as latent geometry the Euclidean one. We set to 2 the dimension of the embedding because the network analysed here is a planar graph, therefore itself is a 2D entity - see Fig. 2.

**Table 1:** Volume and radii of the truncated cone for the Individual 1, calculated as in Eqs. (7) and (9). $$^{( * )}$$$$V = 0$$ indicates null transfer entropy, i.e. absence of interaction

$X$	$Y$	$V$	$\Delta V$	${r_X}$	$\Delta {r_X}$	${r_Y}$	$\Delta {r_Y}$
AS	ASL	0.0711	0.00315	20.21159	2.24584	0.00818	0.00442
AS	ASpOH	0.01572	0.00243	35.4932	1.83947	0.71494	0.35179
AS	ASoOH	0$^{( * )}$	0	29.65423	2.13934	0.46465	0.20335
AS	ASLpOH	0$^{( * )}$	0	28.78377	2.16983	0.02958	0.01066
AS	ASLoOH	0.00734	0.00169	22.00105	2.25597	0.01685	0.00652
ASL	ASpOH	0.00933	0.00179	0.14796	0.01289	3.21513	0.68965
ASL	ASoOH	0.00905	0.00175	0.12975	0.013	2.3137	0.40277
ASL	ASLpOH	0.00867	0.00173	0.06732	0.01135	0.1295	0.0194
ASL	ASLoOH	0.00399	0.00148	0.10725	0.01287	0.04891	0.01044
ASpOH	ASoOH	0.05968	0.00501	5.19208	0.81646	5.27732	0.46076
ASpOH	ASLpOH	0.07513	0.00643	3.75687	0.73229	0.23814	0.02078
ASpOH	ASLoOH	0.09565	0.00729	3.39563	0.70461	0.09287	0.01298
ASoOH	ASLpOH	0.10544	0.00627	2.50242	0.41355	0.17977	0.02078
ASoOH	ASLoOH	0.10869	0.00735	2.21348	0.39589	0.08816	0.01289
ASLpOH	ASLoOH	0.10818	0.00746	0.15052	0.02014	0.07067	0.01193

**Table 2:** Volume and radii of the truncated cone for the Individual 2, calculated as in Eqs. (7) and (9). $$^{( * )}$$$$V = 0$$ indicates null transfer entropy, i.e. absence of interaction

$X$	$Y$	$V$	$\Delta V$	${r_X}$	$\Delta {r_X}$	${r_Y}$	$\Delta {r_Y}$
AS	ASL	0.09853	0.00557	15.22469	1.4123	0.00549	0.00368
AS	ASpOH	0.06892	0.00525	9.59555	1.34368	0.12032	0.06931
AS	ASoOH	0.10948	0.0062	26.78193	0.61782	0.22568	0.08268
AS	ASLpOH	0.01616	0.00203	6.21433	1.17603	0.00063	0.00025
AS	ASLoOH	0.06254	0.00429	12.68739	1.40977	6e-05	2e-05
ASL	ASpOH	0.04025	0.00545	0.06607	0.01144	0.22228	0.08849
ASL	ASoOH	0.11982	0.00743	0.13152	0.01322	0.00021	3e-05
ASL	ASLpOH	0$^{( * )}$	0	0.07147	0.01174	2e-04	0.00011
ASL	ASLoOH	0.02352	0.00363	0.1104	0.013	0.00019	4e-05
ASpOH	ASoOH	0.1139	0.00702	1.52475	0.19422	0.58625	0.1257
ASpOH	ASLpOH	0.07603	0.00471	1.2768	0.18474	0.00257	0.00045
ASpOH	ASLoOH	0.03939	0.00455	1.72077	0.19769	0.00059	6e-05
ASoOH	ASLpOH	0	0	0.77777	0.13902	0.00381	0.00052
ASoOH	ASLoOH	0.06612	0.00397	0.9719	0.14917	0.00058	7e-05
ASLpOH	ASLoOH	0.04891	0.00532	0.00444	0.00055	0.00062	7e-05

**Table 3:** Volume and radii of the truncated cone for the Individual 3, calculated as in Eqs. (7) and (9)

$X$	$Y$	$V$	$\Delta V$	${r_X}$	$\Delta {r_X}$	${r_Y}$	$\Delta {r_Y}$
AS	ASL	0.01409	0.00278	7.89602	1.15697	0.02587	0.00831
AS	ASpOH	0.18894	0.00602	21.98121	0.77727	1.1015	0.28638
AS	ASoOH	0.14413	0.00551	11.83904	1.2386	0.0113	0.00335
AS	ASLpOH	0.22182	0.00787	21.49879	0.83576	0.00581	0.00195
AS	ASLoOH	0.15519	0.00648	14.8091	1.21547	0.00115	0.00051
ASL	ASpOH	0.02149	0.00371	0.06383	0.01181	1.68862	0.33941
ASL	ASoOH	0.0088	0.0023	0.03706	0.00968	0.0141	0.00337
ASL	ASLpOH	0.02205	0.00359	0.06781	0.01255	0.01302	0.00277
ASL	ASLoOH	0.02136	0.00357	0.0314	0.00899	0.0044	0.00093
ASpOH	ASoOH	0.08236	0.00556	0.67428	0.22974	0.03678	0.0048
ASpOH	ASLpOH	0.16705	0.0088	2.02965	0.363	0.02244	0.00335
ASpOH	ASLoOH	0.10716	0.00735	1.18772	0.29582	0.00697	0.00102
ASoOH	ASLpOH	0.09526	0.00632	0.03102	0.00477	0.0053	0.00186
ASoOH	ASLoOH	0.06552	0.0056	0.01263	0.0031	0.00073	4e-04
ASLpOH	ASLoOH	0.12782	0.00696	0.00592	0.00197	0.00504	0.00096

**Table 4:** Heights of the truncated cones for the interactions in Individual 1. $h = 0$ has been obtained from $V = 0$, that denotes absence of interaction (“no interaction” entry in the fifth column). Also in the fifth,“High SE” indicates the cases in which $\Delta h > h$, due to a high standard error on the time series points

$X$	$Y$	$h$	$\Delta h$	
AS	ASL	0.000166	3.7e-05	
AS	ASpOH	1.2e-05	1e-06	
AS	ASoOH	0	0	no interaction
AS	ASLpOH	0	0	no interaction
AS	ASLoOH	1.4e-05	3e-06	
ASL	ASpOH	0.000822	0.000345	
ASL	ASoOH	0.001524	0.000518	
ASL	ASLpOH	0.275789	1.83425	high SE
ASL	ASLoOH	0.199065	3.85797	high SE
ASpOH	ASoOH	0.000693	0.000124	
ASpOH	ASLpOH	0.004762	0.001795	
ASpOH	ASLoOH	0.007705	0.003153	
ASoOH	ASLpOH	0.014929	0.00475	
ASoOH	ASLoOH	0.020342	0.007132	
ASLpOH	ASLoOH	2.698102	4.89419	high SE

**Table 5:** Heights of the truncated cones for the interactions in Individual 2. $h = 0$ has been obtained from $V = 0$, that denotes absence of interaction (“no interaction” entry in the fifth column). Also in the fifth,“High SE” indicates the cases in which $\Delta h > h$, due to a high standard error on the time series points

$X$	$Y$	$h$	$\Delta h$	
AS	ASL	0.000406	7.5e-05	
AS	ASpOH	0.000706	0.000196	
AS	ASoOH	0.000145	7e-06	
AS	ASLpOH	4e-04	0.000151	
AS	ASLoOH	0.000371	8.2e-05	
ASL	ASpOH	0.561439	1.171119	high SE
ASL	ASoOH	6.604244	23.674886	high SE
ASL	ASLpOH	0	0	no interaction
ASL	ASLoOH	1.839596	23.258452	high SE
ASpOH	ASoOH	0.030531	0.006734	
ASpOH	ASLpOH	0.044446	0.012959	
ASpOH	ASLoOH	0.012699	0.002959	
ASoOH	ASLpOH	0	0	no interaction
ASoOH	ASLoOH	0.066804	0.020935	
ASLpOH	ASLoOH	2043.937613	9729268.669899	high SE

**Table 6:** Heights of the truncated cones for the interactions in Individual 3. $h = 0$ has been obtained from $V = 0$, that denotes absence of interaction (“no interaction” entry in the fifth column). Also in the fifth,“High SE” indicates the cases in which $\Delta h > h$, due to a high standard error on the time series points

$X$	$Y$	$h$	$\Delta h$	
AS	ASL	0.000215	6.3e-05	
AS	ASpOH	0.000355	2.5e-05	
AS	ASoOH	0.000981	0.000205	
AS	ASLpOH	0.000458	3.6e-05	
AS	ASLoOH	0.000676	0.000111	
ASL	ASpOH	0.006925	0.00276	
ASL	ASoOH	4.011544	500.514571	high SE
ASL	ASLpOH	3.726363	107.375536	high SE
ASL	ASLoOH	17.837914	2607.265753	high SE
ASpOH	ASoOH	0.163575	0.110694	
ASpOH	ASLpOH	0.038296	0.01363	
ASpOH	ASLoOH	0.072114	0.035985	
ASoOH	ASLpOH	78.776939	4526.141571	high SE
ASoOH	ASLoOH	369.629008	186638.464989	high SE
ASLpOH	ASLoOH	1351.934201	815363.824841	high SE

**Table 7:** Embedding stresses. Data show that the embedding with the least stress is that in Euclidean space. For Individual 1, the difference with the stresses in hyperbolic and spherical space is slightly more pronounced

	Hyperbolic	Euclidean	Spherical
Individual 1	0.13919	0.07544	0.15294
Individual 2	0.93018	0.83815	0.93402
Individual 3	0.87321	0.78206	0.87816

We have recalculated the embedding stress by considering as input matrix

$${D_{ +\rm {error}}} = \{ {h_{ij}} + \Delta {h_{ij}}\} $$

and, for example for Individual 1, we obtained

$$\eqalign{& {\rm{Hyperbolic}}\,{\rm{embedding}}\,{\rm{stress}} = 0.27713 \cr & {\rm{Euclidean}}\,{\rm{embedding}} = 0.18378 \cr & {\rm{Spherical}}\,{\rm{embedding}}\,{\rm{stress}} = 0.28600 \cr} $$

results that confirm what was deduced from Table 7. The experimental error causes an increase in embedding stress, but the geometry that causes the least stress remains Euclidean. Interestingly, while for hyperbolic and spherical geometry the stress is doubled, for Euclidean geometry the stress of the embedding of ${D_{ + {\text{error}}}}$ is more than doubled. Precisely, the stresses in Table 7 and the stress obtained from the embedding of ${D_{ + {\text{error}}}}$ are 1.990969, 2.436032, 1.86996, respectively for hyperbolic, Euclidean and spherical embedding. We interpret this result as an increased sensitivity of embedding in Euclidean space to experimental errors. In view of this result, and the fact that although the Euclidean geometry results in the least stress, the differences between the stresses are not particularly high, we propose that the geometry that best describes the metric space of the network is the hyperbolic geometry (in 2D and with curvature −1). More experimental data would however be necessary for a more in-depth study of the relationship between experimental errors on the data in the distance/dissimilarity matrix and embedding stress. In order to assess whether this result could be due to the embedding method, we calculated the embedding in Euclidean space with another method, the Classical Multidimensional Scaling method [128], obtaining a stress of 0.8585134 for the embedding of $D$ and a stress of 2.277656 for the embedding of ${D_{ + {\text{error}}}}$. Based on this result, we hypothesise that the sensitivity of the embedding in Euclidean space to the experimental errors on the input data is not primarily due to the embedding method, at least on the basis of what we have done in this analysis.

**Fig. 8:**
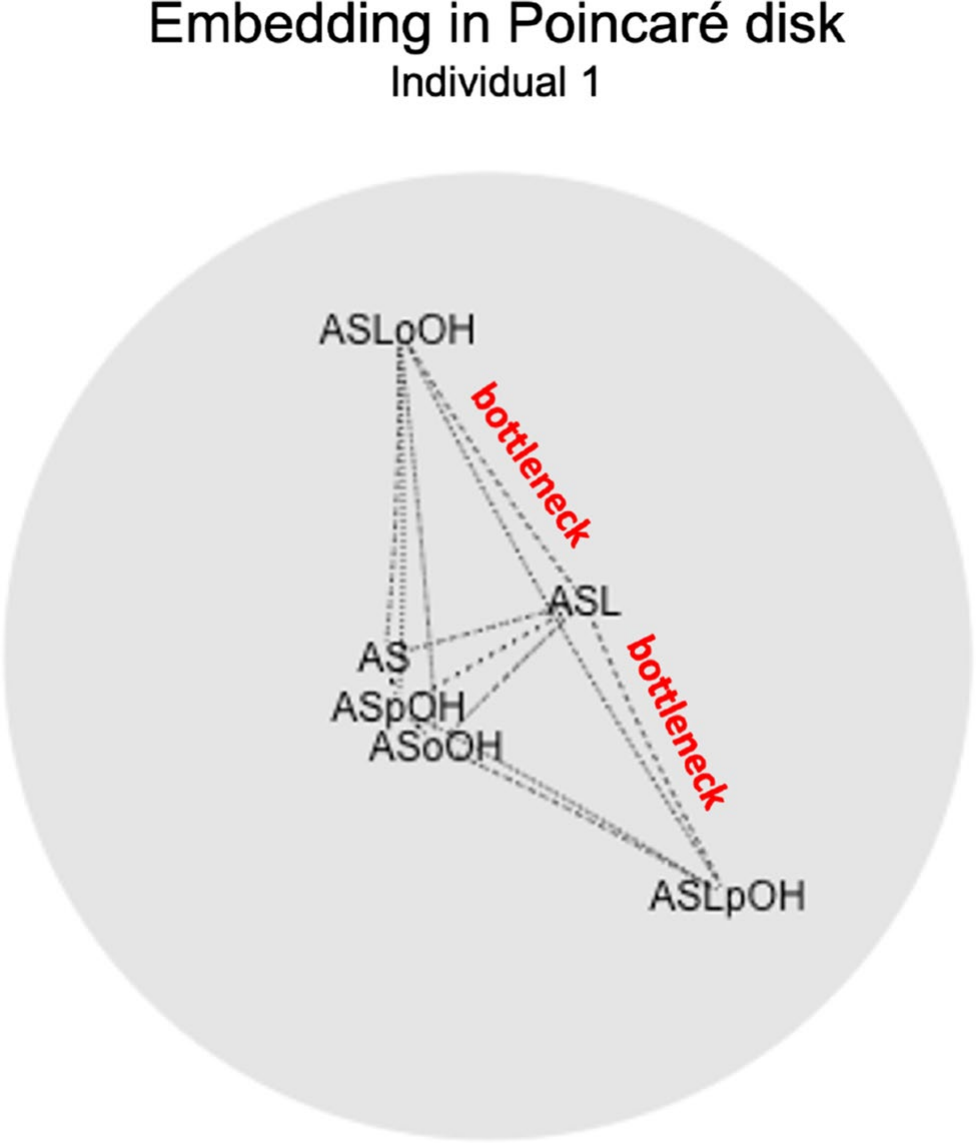
Positioning of the network nodes on the Poincaré disk, a representation of two-dimensional hyperbolic space. The dotted lines connecting two nodes indicate a non-zero value of the matrix D relative to the pair of nodes. The interactions between ASL and ASLoOH and ASLpOH have been identified as bottleneck reactions by our method

**Fig. 9:**
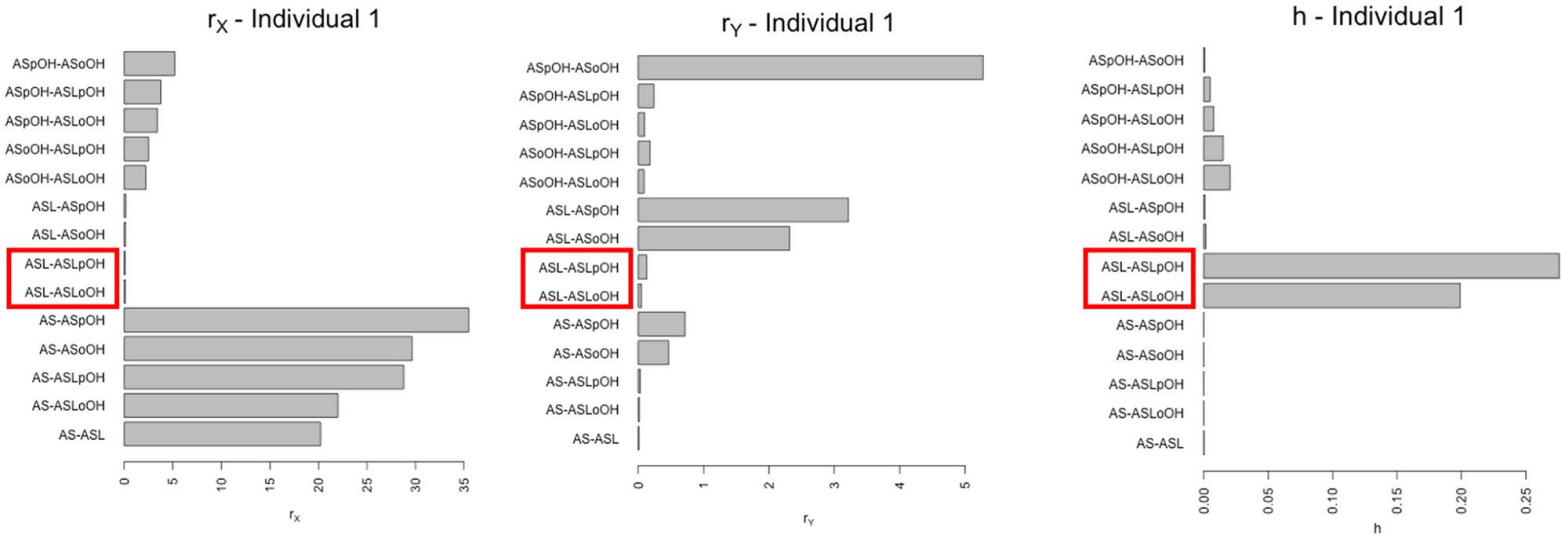
The rectangles in red indicate the interactions that our method classifies as possible bottlenecks

In Figs. 8 and 9, we show the results of the analysis with regard to the data of Individual 1, which, being affected by a smaller standard deviation, made it possible to consider *the majority* of the values of $V$ and $h$ calculated by them to be more accurate. The interactions between ASL and ASLoOH and ASLpOH have been identified as bottleneck reaction by our method (see Figs. 8 and 9).

## Discussion

As we can see in Fig. 2, ASL interacts with ASLoOH and ASLpOH through CYP3A4, which is indeed the major hepatic enzyme metabolizing atorvastatin. The enzymatic conversion of ASL into ASLoOH and ASLpOH are the primary reactions from which the entire metabolism process of atorvastatin begins and whose kinetics are strongly dependent on the concentration of this enzyme and its status (active or inhibited) [129-131]. The distance between ASL and metabolites in the metric space representing the latent geometry of the network reflects the extent to which this reaction (given experimental concentration data and CYP3A4 activity level) is a bottleneck for metabolism. The interaction between AS and CYP3A4 is indeed a complex enzymatic reaction. Recent *in vitro* studies have shown that atorvastatin both activates and inhibits CYP3A4 enzyme. Although there are no well-controlled, longer-term trials that might assess atorvastatin’s inducing effect, certain clinical studies suggest that it inhibits CYP3A4 [131]. The complexity of the reaction and numerous studies on it support the hypothesis that it may in fact be a bottleneck dependent on many factors, such as the activity and concentration of the enzyme, and its possible inhibition by atorvastatin itself. The high value of the standard error on the time series of ASL, ASLpOH and ASLoOH, which then propagates to the estimation of $h$ and $\Delta h$ for the ASL - ASLpOH and ASL - ASLoOH interactions (see Tables 4, 5, and 6), can also be partly explained by the multifactorial dependence of these two bottleneck interactions.

## Conclusions

This study proposes a method for determining the matrix of dissimilarities between nodes calculated from the transfer entropy and its use to determine the metric space of the network and the arrangement of nodes in this space. The analysis of the distance matrix and the arrangement of points in the metric space provide information on possible bottleneck reactions for metabolism. Both the analysis of the distance matrix and the arrangement of nodes in the metric space are necessary for the identification of bottlenecks. The arrangement of nodes in Poincaré space as a finite representation of a hyperbolic space helps classify a distance as unreasonably large for the purposes of effective interaction between nodes (e.g. if nodes are close to the edge of the Poincaré circle). The study also shows how the identification of the latent geometry of a network and consequent bottlenecks is affected by the experimental error or the variance of the input data to the computational procedure. In summary, in addition to the undoubted biological and medical interest in pharmacokinetic networks, and the atorvastatin network in particular, our study is of interest from a mathematical and bioinformatic point of view, as the proposed procedure offers a geometrical interpretation of reaction bottlenecks, which proves to be a versatile tool for the identification of bottlenecks at the system (or network) level and not at the individual reaction level, but which, above all, can be quantified from experimental data (such as time series) that are easier to measure than data such as activation energies and kinetic rate constants [14, 132-143].

## Data Availability

The study uses publicly available experimental data in the supplementary material of Bucher’s paper [107]. All data generated and/or analysed from them during this study are included in this article. The code for node graph embedding is a Python script publicly available in the following GitLab repository. https://gitlab.inf.unibz.it/Paola.Lecca/atorvastatin-pharmacokinetics-analysis The procedure for calculating the dissimilarity matrix from the transfer entropy was implemented in R using the formulae in the Section “Transfer entropy as volume of information” and functions of the library RTransferEntropy [110, 132].

## Appendix: Network latent geometry

The latent geometry of a network is important since it explains the organisational principles and laws of network dynamics [14, 133-136]. The latent geometry of the network is an intrinsic property of a graph, not a modelling of it, and is derivable from the dissimilarity/distance matrix of the graph. The expression *latent geometry of a network* is an abbreviation of the expression ‘geometry of the latent metric space’ of a graph, and the adjective “latent” is justified by the fact that in the usual definition of a network one simply thinks of a set of nodes and arcs rather than a metric space. However, it must be recognised that graph and metric space are in fact the same object, since the topological structure and dynamic properties of the graph (precisely those we are used to considering, such as scale-free properties, the existence of modules, etc.) emerge precisely from the properties of the metric space.

In a network, the likelihood of two nodes being connected by a link (i.e. the interaction propensity) is high if one node is located in close proximity to another node in the latent geometry. Network science, with its wide range of real-world applications, including brain mapping, missing link prediction, and efficient navigation [135], is centred on the latent geometry of a complex network. There hasn’t been much research done to provide a technique to estimate the general unknown latent geometry of complex networks, despite the significant influence topology plays on the structures and operations of complex systems.

We point the reader to some references relevant to the concept of latent geometry in [136-139] and to the recent works of P. Lecca and P. Lecca et al. in [120, 140, 141], where graph embedding techniques are used to determine the latent geometry of a network. Graph embedding is a set of methods that generate graphs’ latent vector representations [137, 142] Although graph embedding can be done at several levels of the graph, the two most common layers are: (i) every node in a network is mapped to a vector via node embedding, and (ii) a graph embedding that converts the entire graph into a vector. In [120, 140, 141], P. Lecca et al. show how it is possible to determine which geometry is most representative of the network’s metric space by means of *nodes* graph embedding aimed at identifying the metric space that produces the least distortion of the input dissimilarity/distance matrix. This is a different use of graph embedding procedures that are normally used to lower the dimensionality of input data. As a side note, it is worth mentioning that often in embedding procedures implemented for this purpose, and especially in many applications of embedding in artificial intelligence techniques, dimension and curvature are not properties of the metric space, but simply hyper-parameters, i.e. quantities that do not necessarily have a special geometric significance [143], but that are useful to guide optimization procedures. The curvature, as long as it is in fact the curvature of the optimal metric space representing the network (and not a hyper-parameter) gives important information about node organisation. For instance, more and closer communities are encouraged by more positive manifold curvature, and repulsion is induced by greater negative curvature.

In this work as in the previous works in [120, 140, 141], we attempt through embedding procedures to understand which of the three types of isotropic spaces (Euclidean, spherical and hyperbolic) most faithfully (i.e. with the least distortion) reproduces the dissimilarity matrix of the graph. In this approach, dimension and curvature are geometric features of the metric space, capable of reflecting dissimilarity and/or distance between nodes. More and closer communities are encouraged by more positive manifold curvature, and repulsion is induced by greater negative curvature.

## Abbreviations

ABCB1: ATP binding cassette subfamily B member 1
ABCC2: ATP binding cassette subfamily C member 2
AS: Atorvastatin
ASL: Atorvastatin lactone
ASLoOH: Ortho-hydroxy-atorvastatin lactone
ASLpOH: Para-hydroxy-atorvastatin lactone
ASoOH: Ortho-hydroxy-atorvastatin acid
ASpOH: Para-hydroxy-atorvastatin acid
CK: Creatine kinase
CYP: Cytochromes P450
CYP2C8: Cytochrome P450 family 2 subfamily C member 8
CYP3A4: Cytochrome P450 family 3 subfamily A member 4
CYP3A5: Cytochrome P450 family 3 subfamily A member 5
DDI: Drug-drug interaction
HMG-CoA: Hydroxymethylglutaryl-coenzyme A reductase inhibitor
MDR1: Multidrug resistance mutation 1
MDR2: Multidrug resistance mutation 2
MRP2: Multidrug resistance-associated protein 2
NTE: Net transfer entropy
OATP: Organic anion-transporting polypeptide
OATP1B1: Organic anion transporting polypeptide 1B1
OATP1B3: Organic anion transporting polypeptide 1B3
OATP2B1: Organic anion transporting polypeptide 2B3
PBPK: Physiologically based pharmacokinetic
PK/PD: Pharmacokinetic/pharmacodynamics
QSAR: Quantitative structure-activity relationship
SLCO1B1: Solute carrier organic anion transporter family member 1B1
SLCO1B1: Solute carrier organic anion transporter family member 1B1
TE: Transfer entropy
UGT: UDP-glucuronosyltransferase
UGT1A3: UDP glucuronosyltransferase family 1 member A3

## References

[1] Namdari A, Li ZS. A review of entropy measures for uncertainty quantification of stochastic processes. Adv Mech Eng. 2019;11(6):168781401985735. 10.1177/1687814019857350.

[2] Mensah GA, Fuster V, Murray CJL, Roth GA. Global burden of cardiovascular diseases and risks collaborators: global burden of cardiovascular diseases and risks, 1990–2022. J Am Coll Cardiol. 2023;82(25):2350–473. 10.1016/j.jacc.2023.11.007. 38092509 10.1016/j.jacc.2023.11.007 PMC7615984

[3] Yu H, Kim PM, Sprecher E, Trifonov V, Gerstein M. The importance of bottlenecks in protein networks: correlation with gene essentiality and expression dynamics. PLOS Comput Biol. 2007;3(4):59. 10.1371/journal.pcbi.0030059. 10.1371/journal.pcbi.0030059 PMC1853125 17447836

[4] Shiraishi F, Suzuki Y. Method for determination of the main bottleneck enzyme in a metabolic reaction network by dynamic sensitivity analysis. Ind Eng Chem Res. 2008;48(1):415–23. 10.1021/ie8005963.

[5] McDermott JE, Taylor RC, Yoon H, Heffron F. Bottlenecks and hubs in inferred networks are important for virulence in salmonella typhimurium. J Comput Biol. 2009;16(2):169–80. 10.1089/cmb.2008.04tt. 19178137 10.1089/cmb.2008.04TT

[6] Dietz K, Jacquot J, Harris G. Hubs and bottlenecks in plant molecular signalling networks. New Phytol. 2010;188(4):919–38. 10.1111/j.1469-8137.2010.03502.x. 20958306 10.1111/j.1469-8137.2010.03502.x

[7] Antoniou D, Schwartz SD. Phase space bottlenecks in enzymatic reactions. J Phys Chem A. 2016;120(3):433–39. 10.1021/acs.jpcb.5b11157. 26756622 10.1021/acs.jpcb.5b11157 PMC4734068

[8] Pang E, Hao Y, Sun Y, Lin K. Differential variation patterns between hubs and bottlenecks in human protein–protein interaction networks. BMC Evol Biol. 2016;16(1). 10.1186/s12862-016-0840-8. 10.1186/s12862-016-0840-8 PMC5131443 27903259

[9] Cheah YE, Xu Y, Sacco SA, Babele PK, Zheng AO, Johnson CH, Young JD. Systematic identification and elimination of flux bottlenecks in the aldehyde production pathway of synechococcus elongatus pcc 7942. Metab Eng. 2020;60:56–65. 10.1016/j.ymben.2020.03.007. 32222320 10.1016/j.ymben.2020.03.007 PMC7217728

[10] Boojari MA, Ghaledari FR, Motamedian E, Soleimani M, Shojaosadati SA. Identifying bottleneck reactions and developing a systemic fed-batch feeding strategy of pichia pastoris through fine-tuning of methanol utilization pathway. 2022. 10.22541/au.165397868.89805525/v1.

[11] Nithya C, Kiran M, Nagarajaram HA. Dissection of hubs and bottlenecks in a protein–protein interaction network. Comput Biol Chem. 2023;102:107802. 10.1016/j.compbiolchem.2022.107802. 36603332 10.1016/j.compbiolchem.2022.107802

[12] Khana DB, Tatli M, Rivera Vazquez J, Weraduwage SM, Stern N, Hebert AS, Angelica Trujillo E, Stevenson DM, Coon JJ, Sharky TD, Amador–Noguez D. Systematic analysis of metabolic bottlenecks in the methylerythritol 4–phosphate (mep) pathway of zymomonas mobilis. mSystems. 2023;8(2). 10.1128/msystems.00092-23. 10.1128/msystems.00092-23 PMC10134818 36995223

[13] Boguñá M, Bonamassa I, De Domenico M, Havlin S, Krioukov D, Serrano MA. Network geometry. Nat Rev Phys. 2021;3(2):114–35. 10.1038/s42254-020-00264-4.

[14] Lecca P, Lombardi G, Latorre RV, Sorio C. How the latent geometry of a biological network provides information on its dynamics: the case of the gene network of chronic myeloid leukaemia. Front Cell Dev Biol. 2023;11. 10.3389/fcell.2023.1235116. 10.3389/fcell.2023.1235116 PMC10704170 38078013

[15] Yang X. Multitissue multiomics systems biology to dissect complex diseases. Trends Mol Med. 2020;26(8):718–28. 10.1016/j.molmed.2020.04.006. 32439301 10.1016/j.molmed.2020.04.006 PMC7395877

[16] Campbell K, Xia J, Nielsen J. The impact of systems biology on bioprocessing. Trends Biotechnol. 2017;35(12):1156–68. 10.1016/j.tibtech.2017.08.011. 28987922 10.1016/j.tibtech.2017.08.011

[17] Yang D, Park SY, Park YS, Eun H, Lee SY. Metabolic engineering of escherichia coli for natural product biosynthesis. Trends Biotechnol. 2020;38(7):745–65. 10.1016/j.tibtech.2019.11.007. 31924345 10.1016/j.tibtech.2019.11.007

[18] Zeng W, Guo L, Xu S, Chen J, Zhou J. High–throughput screening technology in industrial biotechnology. Trends Biotechnol. 2020;38(8):888–906. 10.1016/j.tibtech.2020.01.001. 32005372 10.1016/j.tibtech.2020.01.001

[19] Fukuda A, Kuriya Y, Konishi J, Mutaguchi K, Uemura T, Miura D, Okamoto M. Kinetic modeling and sensitivity analysis for higher ethanol production in self–cloning xylose–using saccharomyces cerevisiae. J Biosci Bioeng. 2019;127(5):563–69. 10.1016/j.jbiosc.2018.10.020. 30482500 10.1016/j.jbiosc.2018.10.020

[20] Voit EO. Biochemical systems theory: a review. ISRN Biomath. 2013;2013:1–53. 10.1155/2013/897658.

[21] Kacser H, Burns JA, Kacser H, Fell DA. The control of flux. Biochem Soc Trans. 1995;23(2):341–66. 10.1042/bst0230341. 7672373 10.1042/bst0230341

[22] Heinrich R, Rapoport TA. A linear steady–state treatment of enzymatic chains. general properties, control and effector strength. Eur J Biochem. 1974;42(1):89–95. 10.1111/j.1432-1033.1974.tb03318.x. 4830198 10.1111/j.1432-1033.1974.tb03318.x

[23] Schoppel K, Trachtmann N, Korzin EJ, Tzanavari A, Sprenger GA, Weuster-Botz D. Metabolic control analysis enables rational improvement of e. coli l–tryptophan producers but methylglyoxal formation limits glycerol–based production. Microb Cell Fact. 2022;21(1). 10.1186/s12934-022-01930-1. 10.1186/s12934-022-01930-1 PMC9531422 36195869

[24] Sriyudthsak K, Uno H, Gunawan R, Shiraishi F. Using dynamic sensitivities to characterize metabolic reaction systems. Math Biosci. 2015;269:153–63. 10.1016/j.mbs.2015.09.002. 26384553 10.1016/j.mbs.2015.09.002

[25] Perumal TM, Wu Y, Gunawan R. Dynamical analysis of cellular networks based on the green’s function matrix. J Theor Biol. 2009;261(2):248–59. 10.1016/j.jtbi.2009.07.037. 19660478 10.1016/j.jtbi.2009.07.037

[26] Perumal TM, Gunawan R. Impulse parametric sensitivity analysis. IFAC Proc. 2011;44(1):9686–90. 10.3182/20110828-6-it-1002.03771.

[27] Perumal TM, Gunawan R. pathPSA: a dynamical pathway–based parametric sensitivity analysis. Ind Eng Chem Res. 2014;53(22):9149–57. 10.1021/ie403277d.

[28] Bröhl T, Lehnertz K. Centrality–based identification of important edges in complex networks. Chaos Interdiscip J Nonlinear Sci. 2019;29(3). 10.1063/1.5081098. 10.1063/1.5081098 30927842

[29] Kumar S, Pauline G, Vindal V. Netva: an r package for network vulnerability and influence analysis. J Biomol Struct Dyn. 2024;1–12. doi:10.1080/07391102.2024.2303607. 10.1080/07391102.2024.2303607 38234040

[30] Ser–Giacomi E, Baudena A, Rossi V, Follows M, Clayton S, Vasile R, López C, Hernández–García E. Lagrangian betweenness as a measure of bottlenecks in dynamical systems with oceanographic examples. Nat Commun. 2021;12(1). 10.1038/s41467-021-25155-9. 10.1038/s41467-021-25155-9 PMC8368092 34400636

[31] Bröhl T, Lehnertz K. A straightforward edge centrality concept derived from generalizing degree and strength. Sci Rep. 2022;12(1). 10.1038/s41598-022-08254-5. 10.1038/s41598-022-08254-5 PMC8922089 35292696

[32] Huang Y, Wang G, Tang Y. Bottleneck Attack Strategies on Complex Communication Networks. Berlin, Heidelberg: Springer; 2010. p. 418–25. 10.1007/978-3-642-14932-0-52.

[33] Sriyudthsak K, Mejia RF, Arita M, Hirai MY. Pasmet: a web–based platform for prediction, modelling and analyses of metabolic systems. Nucleic Acids Res. 2016;44(W1):205–11. 10.1093/nar/gkw415. 10.1093/nar/gkw415 PMC4987946 27174940

[34] Dong S, Zhang Y. Research on method of traffic network bottleneck identification based on max–flow min–cut theorem. In: Proceedings 2011 International Conference on Transportation, Mechanical, and Electrical Engineering (TMEE). IEEE, New York City, USA 2011. 10.1109/tmee.2011.6199586

[35] Chen Y, Yan P, Zheng Z, Chen D. Identifying Traffic Bottleneck in Urban Road Networks via Causal Inference. Cham: Springer; 2021. p. 372–83. 10.1007/978-3-030-68884-4-31.

[36] Li C, Yue W, Mao G, Xu Z. Congestion propagation based bottleneck identification in urban road networks. IEEE Trans Veh Technol. 2020;69(5):4827–41. 10.1109/tvt.2020.2973404.

[37] Qi H, Chen M, Wang D. Recurrent and non–recurrent bottleneck analysis based on traffic state rank distribution. Transportmetrica B Transport Dyn. 2017;7(1):275–94. 10.1080/21680566.2017.1401496.

[38] Lai X, Qiu T, Shui H, Ding D, Ni J. Predicting future production system bottlenecks with a graph neural network approach. J Manuf Syst. 2023;67:201–12. 10.1016/j.jmsy.2023.01.010.

[39] Åkerblom N, Hoseini FS, Haghir Chehreghani M. Online learning of network bottlenecks via minimax paths. Mach Learn. 2022;112(1):131–50. 10.1007/s10994-022-06270-0.

[40] Kempa WM, Paprocka I. A discrete–time queueing model of a bottleneck with an energy–saving mechanism based on setup and shutdown times. Symmetry. 2024;16(1):63. 10.3390/sym16010063.

[41] Duan J, Zeng G, Serok N, Li D, Lieberthal EB, Huang H–J, Havlin S. Spatiotemporal dynamics of traffic bottlenecks yields an early signal of heavy congestions. Nat Commun. 2023;14(1). 10.1038/s41467-023-43591-7. 10.1038/s41467-023-43591-7 PMC10695996 38049413

[42] Serok N, Havlin S, Blumenfeld Lieberthal E. Identification, cost evaluation, and prioritization of urban traffic congestions and their origin. Sci Rep. 2022;12(1). 10.1038/s41598-022-17404-8. 10.1038/s41598-022-17404-8 PMC9338062 35906267

[43] Lighthill MJ, Whitham GB. On kinematic waves ii. a theory of traffic flow on long crowded roads. Proc R Soc Lond A Math Phys Sci. 1955;229(1178):317–45. 10.1098/rspa.1955.0089.

[44] Ni D, Leonard JD. A simplified kinematic wave model at a merge bottleneck. Appl Math Modell. 2005;29(11):1054–72. 10.1016/j.apm.2005.02.008.

[45] Tanimoto J, Hagishima A, Tanaka Y. Study of bottleneck effect at an emergency evacuation exit using cellular automata model, mean field approximation analysis, and game theory. Physica A Stat Mech Appl. 2010;389(24):5611–18. 10.1016/j.physa.2010.08.032.

[46] Biham O, Middleton AA, Levine D. Self–organization and a dynamical transition in traffic–flow models. Phys Rev A. 1992;46(10):6124–27. 10.1103/physreva.46.r6124. 10.1103/physreva.46.r6124 9907993

[47] Kerner BS, Klenov SL, Wolf DE. Cellular automata approach to three–phase traffic theory. J Phys A Math Theor. 2002;35(47):9971–10013. 10.1088/0305-4470/35/47/303.

[48] Pollett PK. Modelling congestion in closed queueing networks. Int Trans Oper Res. 2000;7(4):319–30. 10.1016/S0969-6016(00)00004-6.

[49] Chen C, Skabardonis A, Varaiya P. Systematic identification of freeway bottlenecks. Transp Res Rec. 2004;1867(1):46–52. 10.3141/1867-06.

[50] Li N, Guo R–Y. Simulation of bi–directional pedestrian flow through a bottleneck: cell transmission model. Physica A Stat Mech Appl. 2020;555:124542. 10.1016/j.physa.2020.124542.

[51] Bartlomiejczyk MA, Penson P, Banach M. Worldwide dyslipidemia guidelines. Curr Cardiovasc Risk Rep. 2019;13(2). 10.1007/s12170-019-0597-x.

[52] Cannon CP, Braunwald E, McCabe CH, Rader DJ, Rouleau JL, Belder R, Joyal SV, Hill KA, Pfeffer MA, Skene AM. Intensive versus moderate lipid lowering with statins after acute coronary syndromes. N Engl J Med. 2004;350(15):1495–504. 10.1056/nejmoa040583. 15007110 10.1056/NEJMoa040583

[53] LaRosa JC, Grundy SM, Waters DD, Shear C, Barter P, Fruchart J–C, Gotto AM, Greten H, Kastelein JJP, Shepherd J, Wenger NK. Intensive lipid lowering with atorvastatin in patients with stable coronary disease. N Engl J Med. 2005;352(14):1425–35. 10.1056/nejmoa050461. 15755765 10.1056/NEJMoa050461

[54] Kjekshus J, Dunselman P, Blideskog M, Eskilson C, Hjalmarson M, McMurray JV, Waagstein F, Wedel H, Wessman P, Wikstrand J. A statin in the treatment of heart failure? Controlled rosuvastatin multinational study in heart failure (corona): study design and baseline characteristics. Eur J Heart Failure. 2005;7(6):1059–69. 10.1016/j.ejheart.2005.09.005. 10.1016/j.ejheart.2005.09.005 16227145

[55] The Long-Term Intervention with Pravastatin in Ischaemic Disease (LIPID) Study Group. Prevention of cardiovascular events and death with pravastatin in patients with coronary heart disease and a broad range of initial cholesterol levels. N Engl J Med. 1998;339(19):1349–57. 10.1056/nejm199811053391902. 10.1056/NEJM199811053391902 9841303

[56] Collins R, Reith C, Emberson J, Armitage J, Baigent C, Blackwell L, Blumenthal R, Danesh J, Smith GD, DeMets D, Evans S, Law M, MacMahon S, Martin S, Neal B, Poulter N, Preiss D, Ridker P, Roberts I, Rodgers A, Sandercock P, Schulz K, Sever P, Simes J, Smeeth L, Wald N, Yusuf S, Peto R. Interpretation of the evidence for the efficacy and safety of statin therapy. Lancet. 2016;388(10059):2532–61. 10.1016/s0140-6736(16)31357-5. 27616593 10.1016/S0140-6736(16)31357-5

[57] Reith C, Baigent C, Blackwell L, Emberson J, Spata E, Davies K, Halls H, Holland L, Wilson K, Armitage J, Harper C, Preiss D, Roddick A, Keech A, Simes J, Collins R, Barnes E, Fulcher J, Herrington WG, Kirby A, Mihaylova B, O’Connell R, Amarenco P, Barter P, Betteridge (deceased) DJ, Blazing M, Bosch J, Bowman L, Braunwald E, Cannon CP, Clearfield M, Cobbe S, Colhoun HM, Dahlöf B, Davis B, Lemos J, Downs JR, Durrington PN, Fellström B, Ford I, Franzosi MG, Fuller (deceased) J, Furberg C, Glynn R, Gordon D, Gotto Jr A, Grimm R, Gupta A, Hawkins CM, Hitman GA, Holdaas (deceased) H, Jardine A, Jukema JW, Kastelein JJ, Kean S, Kjekshus J, Knatterud (deceased) G, Knopp (deceased) RH, Koenig W, Koren M, Krane V, Landray M, LaRosa J, Latini R, Lonn E, Lucci D, MacFadyen J, Macfarlane P, MacMahon S, Maggioni A, Marchioli R, Marschner I, Moyé L, Murphy S, Neil A, Nicolis EB, Packard C, Parish S, Pedersen TR, Peto R, Pfeffer M, Poulter N, Pressel S, Probstfield J, Rahman M, Ridker PM, Robertson M, Sacks F, Sattar N, Schmieder R, Serruys PW, Sever P, Shaw (deceased) J, Shepherd (deceased) J, Simpson L, Sleight (deceased) P, Tavazzi L, Tognoni G, Tonkin A, Trompet S, Wanner C, Wedel H, Weis S, Welch KM, White H, Wikstrand J, Wilhelmsen L, Wiviott S, Young R, Yusuf S, Zannad F, Arashi H, Byington R, Clarke R, Flather M, Goldbourt U, Goto S, Hopewell J, Hovingh K, Kearney P, Kitas G, Newman C, Sabatine MS, Schwartz G, Smeeth L, Tobert J, Varigos J, Yamaguchi J. Effect of statin therapy on muscle symptoms: an individual participant data meta–analysis of large–scale, randomised, double–blind trials. Lancet. 2022;400(10355):832–45. 10.1016/S0140-6736(22)01545-8. 10.1016/S0140-6736(22)01545-8 PMC7613583 36049498

[58] Adhyaru BB, Jacobson TA. Safety and efficacy of statin therapy. Nat Rev Cardiol. 2018;15(12):757–69. 10.1038/s41569-018-0098-5. 30375494 10.1038/s41569-018-0098-5

[59] Yebyo HG, Aschmann HE, Kaufmann M, Puhan MA. Comparative effectiveness and safety of statins as a class and of specific statins for primary prevention of cardiovascular disease: a systematic review, meta–analysis, and network meta–analysis of randomized trials with 94, 283 participants. Am Heart J. 2019;210:18–28. 10.1016/j.ahj.2018.12.007. 30716508 10.1016/j.ahj.2018.12.007

[60] Whirl-Carrillo M, Huddart R, Gong L, Sangkuhl K, Thorn CF, Whaley R, Klein TE. An evidence-based framework for evaluating pharmacogenomics knowledge for personalized medicine. Clin Pharmacol Ther. 2021;110(3):563–72. 10.1002/cpt.2350. 34216021 10.1002/cpt.2350 PMC8457105

[61] Alfirevic A, Neely D, Armitage J, Chinoy H, Cooper RG, Laaksonen R, Carr DF, Bloch KM, Fahy J, Hanson A, Yue Q–Y, Wadelius M, Maitland–van Der Zee AH, Voora D, Psaty BM, Palmer CNA, Pirmohamed M. Phenotype standardization for statin–induced myotoxicity. Clin Pharmacol Ther. 2014;96(4):470–76. 10.1038/clpt.2014.121. 24897241 10.1038/clpt.2014.121 PMC4172546

[62] Heckbert SR. Use of administrative data to estimate the incidence of statin–related rhabdomyolysis. JAMA. 2012;307(15):1580. 10.1001/jama.2012.489. 22511681 10.1001/jama.2012.489 PMC3483067

[63] Kee PS, Chin PKL, Kennedy MA, Maggo SDS. Pharmacogenetics of statin–induced myotoxicity. Front Genet. 2020;11. 10.3389/fgene.2020.575678. 10.3389/fgene.2020.575678 PMC7596698 33193687

[64] Lalatović N, Ždralević M, Antunović T, Pantović S. Genetic polymorphisms in abcb1 are correlated with the increased risk of atorvastatin–induced muscle side effects: a cross–sectional study. Sci Rep. 2023;13(1). 10.1038/s41598-023-44792-2. 10.1038/s41598-023-44792-2 PMC10587173 37857778

[65] Wiggins BS, Saseen JJ, Page RL, Reed BN, Sneed K, Kostis JB, Lanfear D, Virani S, Morris PB. Recommendations for management of clinically significant drug–drug interactions with statins and select agents used in patients with cardiovascular disease: a scientific statement from the american heart association. Circulation. 2016;134(21). 10.1161/cir.0000000000000456. 10.1161/CIR.0000000000000456 27754879

[66] Grube M, Kock K, Oswald S, Draber K, Meissner K, Eckel L, Bohm M, Felix S, Vogelgesang S, Jedlitschky G. Organic anion transporting polypeptide 2b1 is a high–affinity transporter for atorvastatin and is expressed in the human heart. Clin Pharmacol Ther. 2006;80(6):607–20. 10.1016/j.clpt.2006.09.010. 17178262 10.1016/j.clpt.2006.09.010

[67] Lau YY, Huang Y, Frassetto L, Benet LZ. Effect of oatp1b transporter inhibition on the pharmacokinetics of atorvastatin in healthy volunteers. Clin Pharmacol Ther. 2006;81(2):194–204. 10.1038/sj.clpt.6100038. 17192770 10.1038/sj.clpt.6100038

[68] Nishimura M, Naito S. Tissue–specific mrna expression profiles of human atp–binding cassette and solute carrier transporter superfamilies. Drug Metab Pharmacokinet. 2005;20(6):452–77. 10.2133/dmpk.20.452. 16415531 10.2133/dmpk.20.452

[69] Hsiang B, Zhu Y, Wang Z, Wu Y, Sasseville V, Yang W-P, Kirchgessner TG. A novel human hepatic organic anion transporting polypeptide (oatp2). J Biol Chem. 1999;274(52):37161–68. 10.1074/jbc.274.52.37161. 10601278 10.1074/jbc.274.52.37161

[70] Chen C, Mireles RJ, Campbell SD, Lin J, Mills JB, Xu JJ, Smolarek TA. Differential interaction of 3–hydroxy–3–methylglutaryl–coa reductase inhibitors with abcb1, abcc2, and oatp1b1. Drug Metab Dispos. 2004;33(4):537–46. 10.1124/dmd.104.002477. 15616150 10.1124/dmd.104.002477

[71] Niessen J, Jedlitschky G, Grube M, Bien S, Schwertz H, Ohtsuki S, Kawakami H, Kamiie J, Oswald S, Starke K, Strobel U, Siegmund W, Rosskopf D, Greinacher A, Terasaki T, Kroemer HK. Human platelets express organic anion–transporting peptide 2b1, an uptake transporter for atorvastatin. Drug Metab Dispos. 2009;37(5):1129–37. 10.1124/dmd.108.024570. 19237515 10.1124/dmd.108.024570

[72] Vildhede A, Karlgren M, Svedberg EK, Wisniewski JR, Lai Y, Norén A, Artursson P. Hepatic uptake of atorvastatin: influence of variability in transporter expression on uptake clearance and drug–drug interactions? Drug Metab Dispos. 2014;42(7):1210–18. 10.1124/dmd.113.056309. 24799396 10.1124/dmd.113.056309

[73] Shitara Y, Sugiyama Y. Pharmacokinetic and pharmacodynamic alterations of 3–hydroxy–3–methylglutaryl coenzyme a (hmg–coa) reductase inhibitors: drug–drug interactions and interindividual differences in transporter and metabolic enzyme functions. Pharmacol Ther. 2006;112(1):71–105. 10.1016/j.pharmthera.2006.03.003. 16714062 10.1016/j.pharmthera.2006.03.003

[74] Li L, Meier PJ, Ballatori N. Oatp2 mediates bidirectional organic solute transport: a role for intracellular glutathione. Mol Pharmacol. 2000;58(2):335–40. 10.1124/mol.58.2.335. 10908301 10.1124/mol.58.2.335

[75] Bogman K, Peyer A, Török M, Küsters E, Drewe J. Hmg-coa reductase inhibitors and p-glycoprotein modulation. Br J Pharmacol. 2001;132(6):1183–92. 10.1038/sj.bjp.0703920. 11250868 10.1038/sj.bjp.0703920 PMC1572659

[76] Sakaeda T, Fujino H, Komoto C, Kakumoto M, Jin J–, Iwaki K, Nishiguchi K, Nakamura T, Okamura N, Okumura K. Effects of acid and lactone forms of eight hmg–coa reductase inhibitors on cyp–mediated metabolism and mdr1–mediated transport. Pharm Res. 2006;23(3):506–12. 10.1007/s11095-005-9371-5. 16388406 10.1007/s11095-005-9371-5

[77] Prueksaritanont T, Subramanian R, Fang X, Ma B, Qiu Y, Lin JH, Pearson PG, Baillie TA. Glucuronidation of statins in animals and humans: a novel mechanism of statin lactonization. Drug Metab Dispos. 2002;30(5):505–12. 10.1124/dmd.30.5.505. 11950779 10.1124/dmd.30.5.505

[78] Schirris TJJ, Ritschel T, Bilos A, Smeitink JAM, Russel FGM. Statin lactonization by uridine 5’–diphospho–glucuronosyltransferases (ugts). Mol Pharmaceut. 2015;12(11):4048–55. 10.1021/acs.molpharmaceut.5b00474. 10.1021/acs.molpharmaceut.5b00474 26412035

[79] Jacobsen W, Kuhn B, Soldner A, Kirchner G, Sewing KF, Kollman PA, Benet LZ, Christians U. Lactonization is the critical first step in the disposition of the 3–hydroxy–3–methylglutaryl–CoA reductase inhibitor atorvastatin. Drug Metab Dispos. 2000;28(11):1369–78. 11038166

[80] Stillemans G, Paquot A, Muccioli GG, Hoste E, Panin N, Åsberg A, Balligand J, Haufroid V, Elens L. Atorvastatin population pharmacokinetics in a real-life setting: influence of genetic polymorphisms and association with clinical response. Clin Transl Sci. 2021;15(3):667–79. 10.1111/cts.13185. 34761521 10.1111/cts.13185 PMC8932751

[81] Tornio A, Pasanen MK, Laitila J, Neuvonen PJ, Backman JT. Comparison of 3-hydroxy-3-methylglutaryl coenzyme a (hmg-coa) reductase inhibitors (statins) as inhibitors of cytochrome p450 2c8. Basic Clin Pharmacol Toxicol. 2005;97(2):104–08. 10.1111/j.1742-7843.2005.pto-134.x. 15998357 10.1111/j.1742-7843.2005.pto_134.x

[82] Gouédard C, Koum–Besson N, Barouki R, Morel Y. Opposite regulation of the human paraoxonase–1 gene pon–1 by fenofibrate and statins. Mol Pharmacol. 2003;63(4):945–56. 10.1124/mol.63.4.945. 12644596 10.1124/mol.63.4.945

[83] Draganov DI, Teiber JF, Speelman A, Osawa Y, Sunahara R, La Du BN. Human paraoxonases (pon1, pon2, and pon3) are lactonases with overlapping and distinct substrate specificities. J Lipid Res. 2005;46(6):1239–47. 10.1194/jlr.m400511-jlr200. 15772423 10.1194/jlr.M400511-JLR200

[84] Draganov DI, Stetson PL, Watson CE, Billecke SS, La Du BN. Rabbit serum paraoxonase 3 (pon3) is a high density lipoprotein–associated lactonase and protects low density lipoprotein against oxidation. J Biol Chem. 2000;275(43):33435–42. 10.1074/jbc.m004543200. 10931838 10.1074/jbc.M004543200

[85] Turner RM, Fontana V, Zhang JE, Carr D, Yin P, FitzGerald R, Morris AP, Pirmohamed M. A genome-wide association study of circulating levels of atorvastatin and its major metabolites. Clin Pharmacol Ther. 2020;108(2):287–97. 10.1002/cpt.1820. 32128760 10.1002/cpt.1820

[86] Türkmen D, Masoli JAH, Kuo C, Bowden J, Melzer D, Pilling LC. Statin treatment effectiveness and the slco1b1*5 reduced function genotype: long-term outcomes in women and men. Br J Clin Pharmacol. 2022;88(7):3230–40. 10.1111/bcp.15245. 35083771 10.1111/bcp.15245 PMC9305522

[87] Gao Y, Zhang L–, Fu Q. Cyp3a4*1g polymorphism is associated with lipid-lowering efficacy of atorvastatin but not of simvastatin. Eur J Clin Pharmacol. 2008;64(9):877–82. 10.1007/s00228-008-0502-x. 18528690 10.1007/s00228-008-0502-x

[88] Kim S, Seo JD, Yun Y–M, Kim H, Kim T–E, Lee T, Lee T–R, Lee JH, Cho E–H, Ki C–S. Pharmacokinetics and genetic factors of atorvastatin in healthy korean subjects. Front Genet. 2022;13. 10.3389/fgene.2022.836970. 10.3389/fgene.2022.836970 PMC9160745 35664336

[89] Vanwong N, Tipnoppanon S, Na Nakorn C, Srisawasdi P, Rodcharoen P, Medhasi S, Chariyavilaskul P, Siwamogsatham S, Vorasettakarnkij Y, Sukasem C. Association of drug-metabolizing enzyme and transporter gene polymorphisms and lipid-lowering response to statins in thai patients with dyslipidemia. Pharmacogenomics Pers Med. 2022;15:119–30. 10.2147/pgpm.s346093. 10.2147/PGPM.S346093 PMC8860396 35210819

[90] Duarte JD, Cavallari LH. Pharmacogenetics to guide cardiovascular drug therapy. Nat Rev Cardiol. 2021;18(9):649–65. 10.1038/s41569%96021%9600549%96w. 10.1038/s41569-021-00549-w PMC8364496 33953382

[91] Verma J, Khedkar V, Coutinho E. 3d-qsar in drug design– a review. Curr Top Med Chem. 2010;10(1):95–115. 10.2174/156802610790232260. 19929826 10.2174/156802610790232260

[92] Tropsha A, Isayev O, Varnek A, Schneider G, Cherkasov A. Integrating qsar modelling and deep learning in drug discovery: the emergence of deep qsar. Nat Rev Drug Discovery. 2023. doi:10.1038/s41573-023-00832-0. 10.1038/s41573-023-00832-0 38066301

[93] Wang Z, Cheng L, Kai Z, Wu F, Liu Z, Cai M. Molecular modeling studies of atorvastatin analogues as hmgr inhibitors using 3d-qsar, molecular docking and molecular dynamics simulations. Bioorg Med Chem Lett. 2014;24(16):3869–76. 10.1016/j.bmcl.2014.06.055. 25022881 10.1016/j.bmcl.2014.06.055

[94] Guillén D, Millán O, Brunet M. In vitro studies of the immunomodulatory effects of statins alone and in combination with immunosuppressive drugs. Eur J Inflammation. 2011;9(2):117–24. 10.1177/1721727X1100900205.

[95] Arayne MS, Sultana N, Rizvi SBS, Haroon U. In vitro drug interaction studies of atorvastatin with ciprofloxacin, gatifloxacin, and ofloxacin. Med Chem Res. 2009;19(8):717–31. 10.1007/s00044-009-9225-5.

[96] Shaker MA, Elbadawy HM, Shaker MA. Improved solubility, dissolution, and oral bioavailability for atorvastatin-pluronic solid dispersions. Int J Pharm. 2020;574:118891. 10.1016/j.ijpharm.2019.118891. 31786357 10.1016/j.ijpharm.2019.118891

[97] Reig–López J, García–Arieta A, Mangas–Sanjuán V, Merino–Sanjuán M. Current evidence, challenges, and opportunities of physiologically based pharmacokinetic models of atorvastatin for decision making. Pharmaceutics. 2021;13(5):709. 10.3390/pharmaceutics13050709. 34068030 10.3390/pharmaceutics13050709 PMC8152487

[98] Poli A. Atorvastatin: pharmacological characteristics and lipid-lowering effects. Drugs. 2007;67(Supplement 1):3–15. 10.2165/00003495-200767001-00002. 17910517 10.2165/00003495-200767001-00002

[99] Maklakova SY, Lopukhov AV, Khudyakov AD, Kovalev SV, Mazhuga MP, Chepikova OE, Zamyatnin AA, Majouga AG, Klyachko NL, Beloglazkina EK. Design and synthesis of atorvastatin derivatives with enhanced water solubility, hepatoselectivity and stability. RSC Med Chem. 2023;14(1):56–64. 10.1039/d2md00119e. 36760736 10.1039/d2md00119e PMC9890652

[100] Danhof M, Alvan G, Dahl SG, Kuhlmann J, Paintaud G. Mechanism-based pharmacokinetic-pharmacodynamic modeling—a new classification of biomarkers. Pharm Res. 2005;22(9):1432–37. 10.1007/s11095-005-5882-3. 16132354 10.1007/s11095-005-5882-3

[101] Jamei M, Marciniak S, Edwards D, Wragg K, Feng K, Barnett A, Rostami–Hodjegan A. The simcyp population based simulator: architecture, implementation, and quality assurance. Silico Pharmacol. 2013;1(1). 10.1186/2193-9616-1-9. 10.1186/2193-9616-1-9 PMC4230310 25505654

[102] Zhang T. Physiologically based pharmacokinetic modeling of disposition and drug-drug interactions for atorvastatin and its metabolites. Eur J Pharm Sci. 2015;77:216–29. 10.1016/j.ejps.2015.06.019. 26116278 10.1016/j.ejps.2015.06.019

[103] Li S, Yu Y, Jin Z, Dai Y, Lin H, Jiao Z, Ma G, Cai W, Han B, Xiang X. Prediction of pharmacokinetic drug-drug interactions causing atorvastatin-induced rhabdomyolysis using physiologically based pharmacokinetic modelling. Biomed Pharmacother. 2019;119:109416. 10.1016/j.biopha.2019.109416. 31518878 10.1016/j.biopha.2019.109416

[104] Duan P, Zhao P, Zhang L. Physiologically based pharmacokinetic (pbpk) modeling of pitavastatin and atorvastatin to predict drug–drug interactions (ddis). Eur J Drug Metab Pharmacokinet. 2016;42(4):689–705. 10.1007/s13318-016-0383-9. 10.1007/s13318-016-0383-9 27858342

[105] Morse BL, Alberts JJ, Posada MM, Rehmel J, Kolur A, Tham LS, Loghin C, Hillgren KM, Hall SD, Dickinson GL. Physiologically-based pharmacokinetic modeling of atorvastatin incorporating delayed gastric emptying and acid-to-lactone conversion. CPT Pharmacometrics Syst Pharmacol. 2019;8(9):664–75. 10.1002/psp4.12447. 31250974 10.1002/psp4.12447 PMC6765700

[106] Reig–López J, Merino–Sanjuan M, García–Arieta A, Mangas–Sanjuán V. A physiologically based pharmacokinetic model for open acid and lactone forms of atorvastatin and metabolites to assess the drug-gene interaction with slco1b1 polymorphisms. Biomed Pharmacother. 2022;156:113914. 10.1016/j.biopha.2022.113914. 36306592 10.1016/j.biopha.2022.113914

[107] Bucher J, Riedmaier S, Schnabel A, Marcus K, Vacun G, Weiss TS, Thasler WE, Nüssler AK, Zanger UM, Reuss M. A systems biology approach to dynamic modeling and inter-subject variability of statin pharmacokinetics in human hepatocytes. BMC Syst Biol. 2011;5(1). 10.1186/1752-0509-5-66. 10.1186/1752-0509-5-66 PMC3117731 21548957

[108] Grund F. Forsythe, g. e./malcolm, m. a./moler, c. b., computer methods for mathematical computations. englewood cliffs, new jersey 07632. prentice hall, inc., 1977. xi, 259 s. ZAMM J Appl Math Mech/Z Angew Math Mech. 1979;59(2):141–42. 10.1002/zamm.19790590235.

[109] Basak US, Sattari S, Hossain M, Horikawa K, Komatsuzaki T. Transfer entropy dependent on distance among agents in quantifying leader-follower relationships. Biophys Physicobiology. 2021;18:131–44. 10.2142/biophysico.bppb-v18.015. 10.2142/biophysico.bppb-v18.015 PMC8214925 34178564

[110] Behrendt S, Dimpfl T, Peter FJ, Zimmermann DJ. Rtransferentropy — quantifying information flow between different time series using effective transfer entropy. SoftwareX. 2019;10:100265. 10.1016/j.softx.2019.100265.

[111] Yao C–Z, Li H–Y. Effective transfer entropy approach to information flow among EPU, investor sentiment and stock market. Front Phys. 2020;8. 10.3389/fphy.2020.00206.

[112] Prokopenko M, Lizier J, Price D. On thermodynamic interpretation of transfer entropy. Entropy. 2013;15(2):524–43. 10.3390/e15020524.

[113] Imaizumi T, Umeki N, Yoshizawa R, Obuchi T, Sako Y, Kabashima Y. Assessing transfer entropy from biochemical data. Phys Rev E. 2022;105(3). 10.1103/physreve.105.034403. 10.1103/PhysRevE.105.034403 35428091

[114] Vicente R, Wibral M, Lindner M, Pipa G. Transfer entropy—a model-free measure of effective connectivity for the neurosciences. J Comput Neurosci. 2010;30(1):45–67. 10.1007/s10827-010-0262-3. 20706781 10.1007/s10827-010-0262-3 PMC3040354

[115] Shorten DP, Spinney RE, Lizier JT. Estimating transfer entropy in continuous time between neural spike trains or other event-based data. PLOS Comput Biol. 2021;17(4):1008054. 10.1371/journal.pcbi.1008054. 10.1371/journal.pcbi.1008054 PMC8084348 33872296

[116] Ursino M, Ricci G, Magosso E. Transfer entropy as a measure of brain connectivity: a critical analysis with the help of neural mass models. Front Comput Neurosci. 2020;14. 10.3389/fncom.2020.00045. 10.3389/fncom.2020.00045 PMC7292208 32581756

[117] Schreiber T. Measuring information transfer. Phys Rev Lett. 2000;85(2):461–64. 10.1103/physrevlett.85.461. 10991308 10.1103/PhysRevLett.85.461

[118] Behrendt S, Dimpfl T, Peter FJ, Zimmermann DJ. RTransferEntropy —— cran.r–project.org. 2023. https://cran.r-project.org/web/packages/RTransferEntropy/vignettes/transfer-entropy.html. 4 Apr 2024.

[119] Lecca P. Stochastic chemical kinetics. Biophys Rev. 2013;5(4):323–45. 10.1007/s12551-013-0122-2. 28510113 10.1007/s12551-013-0122-2 PMC5425731

[120] Lecca P. Uncovering the geometry of protein interaction network: the case of SARS–CoV–2 protein interactome. In: Vlachos D (ed.) AIP Conference Proceedings, 11th International Conference on Mathematical Modeling in Physical Sciences, IC–MSQUARE 2022, vol. 1. AIP Publishing, Melville, NY USA (2023). 10.1063/5.0163052.

[121] Lecca P, Re A. Checking for non-euclidean latent geometry of biological networks. In: 2022 IEEE International Conference on Bioinformatics and Biomedicine (BIBM), Las Vegas, NV, USA, pp. 2526–35. IEEE, NY, USA 2022. 10.1109/bibm55620.2022.9995274.

[122] Lecca P, Re A, Lombardi G, Latorre RV, Sorio C. Graph Embedding of Chronic Myeloid Leukaemia K562 Cells Gene Network Reveals a Hyperbolic Latent Geometry. Singapore: Springer; 2023. p. 979–91. 10.1007/978-981-99-3091-3_80.

[123] Blumenthal LM. Theory and Applications of Distance Geometry. NY, USA: Chelsea Publishing Company.

[124] Schoenberg IJ. Remarks to Maurice Frechet’s article sur la definition axiomatique d’une classe d’espace distances vectoriellement applicable sur l’espace de hilbert. Ann Math. 1935;36(3):724. 10.2307/1968654.

[125] Begelfor E, Werman M. The world is not always flat or learning curved manifolds 2006. https://api.semanticscholar.org/CorpusID:9928027

[126] Zack GW, Rogers WE, Latt SA. Automatic measurement of sister chromatid exchange frequency. J Histochem Cytochem. 1977;25(7):741–53. 10.1177/25.7.70454. 70454 10.1177/25.7.70454

[127] Glasbey CA. An analysis of histogram-based thresholding algorithms. Cvgip. 1993;55(6):532–37. 10.1006/cgip.1993.1040.

[128] Gower JC. Principal Coordinates Analysis. Wiley; 2014. 10.1002/9781118445112.stat05670.

[129] Park J–E, Kim K–B, Bae SK, Moon B–S, Liu K–H, Shin J–G. Contribution of cytochrome p450 3a4 and 3a5 to the metabolism of atorvastatin. Xenobiotica. 2008;38(9):1240–51. 10.1080/00498250802334391. 18720283 10.1080/00498250802334391

[130] Filppula AM, Hirvensalo P, Parviainen H, Ivaska VE, Lönnberg KI, Deng F, Viinamäki J, Kurkela M, Neuvonen M, Niemi M. Comparative hepatic and intestinal metabolism and pharmacodynamics of statins. Drug Metab Dispos. 2021;49(8):658–67. 10.1124/dmd.121.000406. 34045219 10.1124/dmd.121.000406

[131] Willrich MAV, Rodrigues AC, Cerda A, Genvigir FDV, Arazi SS, Dorea EL, Bernik MMS, Bertolami MC, Faludi A, Largura A, Baudhuin LM, Bryant SC, Hirata MH, Hirata RDC. Effects of atorvastatin on cyp3a4 and cyp3a5 mrna expression in mononuclear cells and cyp3a activity in hypercholeresterolemic patients. Clin Chim Acta. 2013;421:157–63. 10.1016/j.cca.2013.03.007. 23501331 10.1016/j.cca.2013.03.007

[132] Zimmermann D, Behrendt S, Dimpfl T, Peter F. RTransferEntropy: measuring information flow between time series with Shannon and Renyi transfer entropy — CRAN.R-project.org. 2023. https://CRAN.R-project.org/package=RTransferEntropy. 15 Feb 2024.

[133] Wu Z, Menichetti G, Rahmede C, Bianconi G. Emergent complex network geometry. Sci Rep. 2015;5(1). 10.1038/srep10073. 10.1038/srep10073 PMC4434965 25985280

[134] Mulder D, Bianconi G. Network geometry and complexity. J Stat Mech. 2018;173(3–4):783–805. 10.1007/s10955-018-2115-9.

[135] Jhun B. Topological analysis of the latent geometry of a complex network. Chaos Interdiscip J Nonlinear Sci. 2022;32(1):013116. 10.1063/5.0073107. 10.1063/5.0073107 35105131

[136] Lubold S, Chandrasekhar AG, McCormick TH. Identifying the latent space geometry of network models through analysis of curvature. J R Stat Soc Ser B Stat Methodol. 2023;85(2):240–92. 10.1093/jrsssb/qkad002.

[137] Smith AL, Asta DM, Calder CA. The geometry of continuous latent space models for network data. Stat Sci. 2019;34(3). 10.1214/19-sts702. 10.1214/19-sts702 PMC7682928 33235407

[138] Salter-Townshend M, McCormick TH. Latent space models for multiview network data. Ann Appl Stat. 2017;11(3):1217–44. http://www.jstor.org/stable/26362225 29721127 10.1214/16-AOAS955 PMC5927604

[139] Cape J. Spectral analysis of networks with latent space dynamics and signs. Stat. 2021;10(1). 10.1002/sta4.381.

[140] Lecca P, Lecca M. Graph embedding and geometric deep learning relevance to network biology and structural chemistry. Front Artif Intell. 2023;6. 10.3389/frai.2023.1256352. 10.3389/frai.2023.1256352 PMC10687447 38035201

[141] Paola L, Angela R, Giulia L, Valeria LR, Claudio S. Graph embedding of chronic myeloid leukaemia K562 cells gene network reveals a hyperbolic latent geometry, in Proceedings of Eighth International Congress on Information and Communication Technology, ed. by Xin-She Y, Simon SR, Dey N, Amit J (Springer Nature Singapore, Singapore, 2023), pp. 979–91

[142] Cai H, Zheng VW, Chang KCC. A comprehensive survey of graph embedding: problems, techniques, and applications. IEEE Trans Knowl Data Eng. 2018;30(9):1616–37. 10.1109/tkde.2018.2807452.

[143] Gu W, Tandon A, Ahn YY, Radicchi F. Principled approach to the selection of the embedding dimension of networks. Nat Commun. 2021;12(1). 10.1038/s41467-021-23795-5. 10.1038/s41467-021-23795-5 PMC8213704 34145234

